# Species of *Characidotrema* Paperna & Thurston, 1968 (Monogenea: Dactylogyridae) from fishes of the Alestidae (Characiformes) in Africa: new species, host-parasite associations and first insights into the phylogeny of the genus

**DOI:** 10.1186/s13071-019-3580-y

**Published:** 2019-07-26

**Authors:** Eva Řehulková, Maria Lujza Kičinjaová, Zuheir N. Mahmoud, Milan Gelnar, Mária Seifertová

**Affiliations:** 1 Department of Botany and Zoology, Faculty of Science, Masaryk University, Kotlářská 2, 611 37 Brno, Czech Republic; 2 Department of Zoology, Faculty of Science, University of Khartoum, Khartoum, Sudan

**Keywords:** Monogenea, Dactylogyridae, *Characidotrema*, Alestidae, *Brycinus*, Africa, Diversity, DNA

## Abstract

**Background:**

African tetras (Alestidae) belonging to *Brycinus* Valenciennes are known to be parasitized with monogeneans attributed to two genera, *Annulotrema* Paperna & Thurston, 1969 and *Characidotrema* Paperna & Thurston, 1968 (Dactylogyridae). During a survey of monogeneans parasitizing alestids, species of *Characidotrema* were collected in Cameroon, D. R. Congo, Senegal, South Africa, Sudan and Zimbabwe. This paper provides new morphological data and the first molecular analysis broadening our knowledge on the diversity of these parasites.

**Results:**

Seven species (four known and three new) of *Characidotrema* are reported from two species of *Brycinus*: *C. auritum* n. sp. and *C. vespertilio* n. sp. from *B. imberi* (Peters); and *C. brevipenis* Paperna, 1969, *C. nursei* Ergens, 1973, *C. pollex* n. sp., *C. spinivaginus* (Paperna, 1973) and *C. zelotes* Kritsky, Kulo & Boeger, 1987 from *B. nurse* (Rüppell). Species identification was based on morphological analysis of the sclerotized structures supported by nuclear ribosomal DNA (partial *18S* rDNA, ITS1, and *28S* rDNA) sequence data. Morphological analysis confirmed that the most apparent character distinguishing species in the genus is the morphology of the male copulatory organ and vagina. Observations on the haptoral sclerotized elements of these parasites by means of phase contrast microscopy revealed the presence of a sheath-like structure relating to the ventral anchor, a feature that supplements the generic diagnosis of *Characidotrema*. Maximum Likelihood and Bayesian analyses of the large subunit (*28S*) rDNA sequences recovered *Characidotrema* species isolated from the two *Brycinus* hosts as monophyletic, and indicated a closer relationship of this group to monogeneans parasitizing African cyprinids (*Dactylogyrus* spp.) and cichlids (species of *Cichlidogyrus* Paperna, 1960, *Scutogyrus* Pariselle & Euzet, 1995, and *Onchobdella* Paperna, 1968) than to those from catfishes (species of *Quadriacanthus* Paperna, 1961, *Schilbetrema* Paperna & Thurston, 1968 and *Synodontella* Dossou & Euzet, 1993). The overall agreement between the morphological diversification of the MCOs and the molecular tree observed in this study indicates that significant phylogenetic signals for clarifying relationships among species of *Characidotrema* are present in the characteristics of the MCO.

**Conclusions:**

It seems that intra-host speciation is an important force shaping the present distribution and diversity of *Characidotrema* but further studies are necessary to confirm this hypothesis and assess questions related to the phylogeny of these parasites. To identify potential co-speciation events, co-phylogenetic analyses of these monogeneans and their alestid hosts are required.

## Background

African tetras (Alestidae) belonging to *Brycinus* Valenciennes are known to be parasitized by species of *Annulotrema* Paperna & Thurston, 1969 (17 spp.) and *Characidotrema* Paperna & Thurston, 1968 (9 spp.) (see [1, 2]). Species of *Characidotrema* are easily differentiated from those of *Annulotrema* by possessing a robust body with a poorly developed haptor and highly modified ventral anchor-bar complex. The presence of a ventral anchor with a diagonally truncate or scoop-shaped point is an unusual feature that is unique among African dactylogyrids. However, a similarly modified ventral anchor also occurs in species of *Jainus* Mizelle, Kritsky & Crane, 1968, a genus parasitizing characiform fishes in South America. In fact, Kritsky et al. [3] resurrected *Characidotrema*, so far considered as a junior synonym of *Jainus* by Paperna [4]. Although both genera possess similar characteristics and parasitize fishes of the Characiformes, Kritsky et al. [3] emended the diagnosis of *Characidotrema* and clearly differentiated its species from those of *Jainus* by the comparative morphology of the haptoral sclerites and the presence of dextral vagina (*vs* sinistral vagina in *Jainus*).

To date, species of *Characidotrema* have been recorded on the gills of four alestid genera (*Alestes* Müller & Troschel, *Brycinus* Valenciennes, *Phenacogrammus* Eigenmann and *Hemigrammopetersius* Pellegrin) from seven African countries: Cameroon, Egypt, Ghana, Kenya, Tanzania, Togo and Uganda [5–11] (Table 1).

**Table 1:** Species of *Characidotrema* recorded on the gills of African tetras (Alestidae)

*Characidotrema* spp.	Host species	Country	Locality	Reference
*C. auritum* n. sp.	*Brycinus imberi*	South Africa	Pongola River	Present study
		Zimbabwe	Lake Kariba	Present study
*C. brevipenis* Paperna, 1969	*Alestes baremoze*	Ghana	Lake Volta	[7]
	*Brycinus nurse*	Ghana	Lake Volta	[7]
		Senegal	Gambia River	Present study
			Mare de Simenti	Present study
		Sudan	Blue Nile	Present study
			White Nile	Present study
	*Brycinus* cf. *nurse*	Togo	Mono River	[3]
*C. elongata* Paperna & Thurston, 1968^a^	*Brycinus jacksonii*	Uganda	Lake Victoria	[5]
	*Brycinus leuciscus*	Ghana	Lake Volta	[7]
*C. nursei* Ergens, 1973	*Alestes dentex* (!)^b^	Egypt (!)^b^	–	[10]
	*Brycinus leuciscus* (!)^b^	Ghana (!)^b^	Lake Volta (!)^b^	[8]
	*Brycinus nurse*	Egypt	Nile River	[9]
		Sudan	Blue Nile	Present study
			White Nile	Present study
		Uganda	Lake Albert	[4]
*C. nzoiae* (Paperna, 1979)	*Brycinus jacksonii*	Kenya	Nzoia River	[8]
*C. pollex* n. sp.	*Brycinus nurse*	Senegal	Gambia River	Present study
		Sudan	Blue Nile	Present study
			White Nile	Present study
*C. regia* Birgi, 1988	*Brycinus kingsleyae*	Cameroon	Nyong River	[11]
*C. ruahae* (Paperna, 1979)	*Brycinus imberi*	Tanzania	Ruaha River	[8]
*C. spinivaginus* (Paperna, 1973)	*Brycinus nurse*	Ghana	Lake Volta	[4]
		Uganda	Lake Albert	[4]
		Senegal	Gambia River	Present study
		Sudan	Blue Nile	Present study
			White Nile	Present study
*C. spiropenis* Birgi, 1988	*Hemigrammopetersius pulcher*	Cameroon	Nyong River	[11]
	*Phenacogrammus major*	Cameroon	Nyong River	[11]
	*Phenacogrammus urotaenia*	Cameroon	Nyong River	[11]
*C. undifera* Kritsky, Kulo & Boeger, 1987	*Brycinus* cf. *nurse*	Togo	Mono River	[3]
*C. vespertilio* n. sp.	*Brycinus imberi*	Cameroon	Boumba River	Present study
		DR Congo	Lindi River	Present study
*C. zelotes* Kritsky, Kulo & Boeger, 1987	*Brycinus nurse*	Senegal	Gambia River	Present study
		Sudan	White Nile	Present study
	*Brycinus* cf. *nurse*	Togo	Mono River	[3]

^a^Type-species

^b^(!) Erroneous record

During our investigation of gill parasites of alestid fishes from Cameroon, D. R. Congo, Senegal, South Africa, Sudan and Zimbabwe, four previously described and three new species of *Characidotrema* were collected from the gills of *Brycinus imberi* (Peters) and *B. nurse* (Rüppell). Herein, all species found are reported and described using two complementary approaches: a morphological study of the hard, sclerotized structures (i.e. those of the haptor and the distal parts of the female and male reproductive systems), and a molecular study using ribosomal DNA sequences (partial *18S*-ITS1 and partial *28S* rDNA sequences). In addition, to study the placement of *Characidotrema* among other genera of dactylogyrids parasitizing African freshwater fishes, phylogenetic analyses based on sequences of *28S* ribosomal RNA gene were performed.

## Methods

### Fish collection

Fish hosts were collected by beach seine net or hook-and-line or purchased at local fish markets in six African countries: Cameroon, D. R. Congo, Senegal, South Africa, Sudan and Zimbabwe (for localities and coordinates, see Table 2) during the period 2005–2017. Host names recorded here are those provided in FishBase [12]; the names used in the original descriptions are retained in parentheses as synonyms. Live fishes (only the ones captured by net or hook-and-line) were kept in aerated holding tanks until processed for parasitological examination; fishes were sacrificed by severing the spinal cord.

**Table 2:** Localities from which alestid species were collected

Locality	Coordinates	Year of collection
Cameroon, Boumba River	03°18′44.28″N, 14°04′40.79″E	2017
DR Congo, Lindi River, Ndulo Island	00°34′38.99″N, 25°07′11.39″E	2014
Senegal, Gambia River, Simenti	13°01′23.40″N, 13°17′21.00″W	2005–2008
Senegal, Oxbow Mare, Simenti	13°01′47.39″N, 13°17′35.99″W	2005–2008
South Africa, Pongola River, Broken Bridge	26°52′57.71″S, 32°18′40.68″E	2017
Sudan, Blue Nile, Sennar	13°32′31.09″N, 33°37′15.79″E	2010, 2014
Sudan, White Nile, Kosti	13°10′18.58″N, 32°40′19.24″E	2010, 2014
Zimbabwe, Lake Kariba	16°04′50.99″S, 28°52′04.00″E	2012

### Parasite collection and identification

Monogeneans were collected from the gills of freshly killed fishes using fine needles and processed as in Francová et al. [13]. Parasite specimens used for morphological study of the sclerotized structures (the haptoral and reproductive hard parts) were completely flattened using coverslip pressure and fixed with a mixture of glycerine and ammonium picrate (GAP). Specimens used for DNA analyses were bisected using fine needles under a dissecting microscope, and subsequently, the posterior half of the body was placed in a 1.5 ml Eppendorf tube with 96% ethanol for genomic DNA extraction. The anterior body part containing the male copulatory organ was completely flattened under coverslip pressure and fixed with GAP for species identification.

**Fig. 1:**
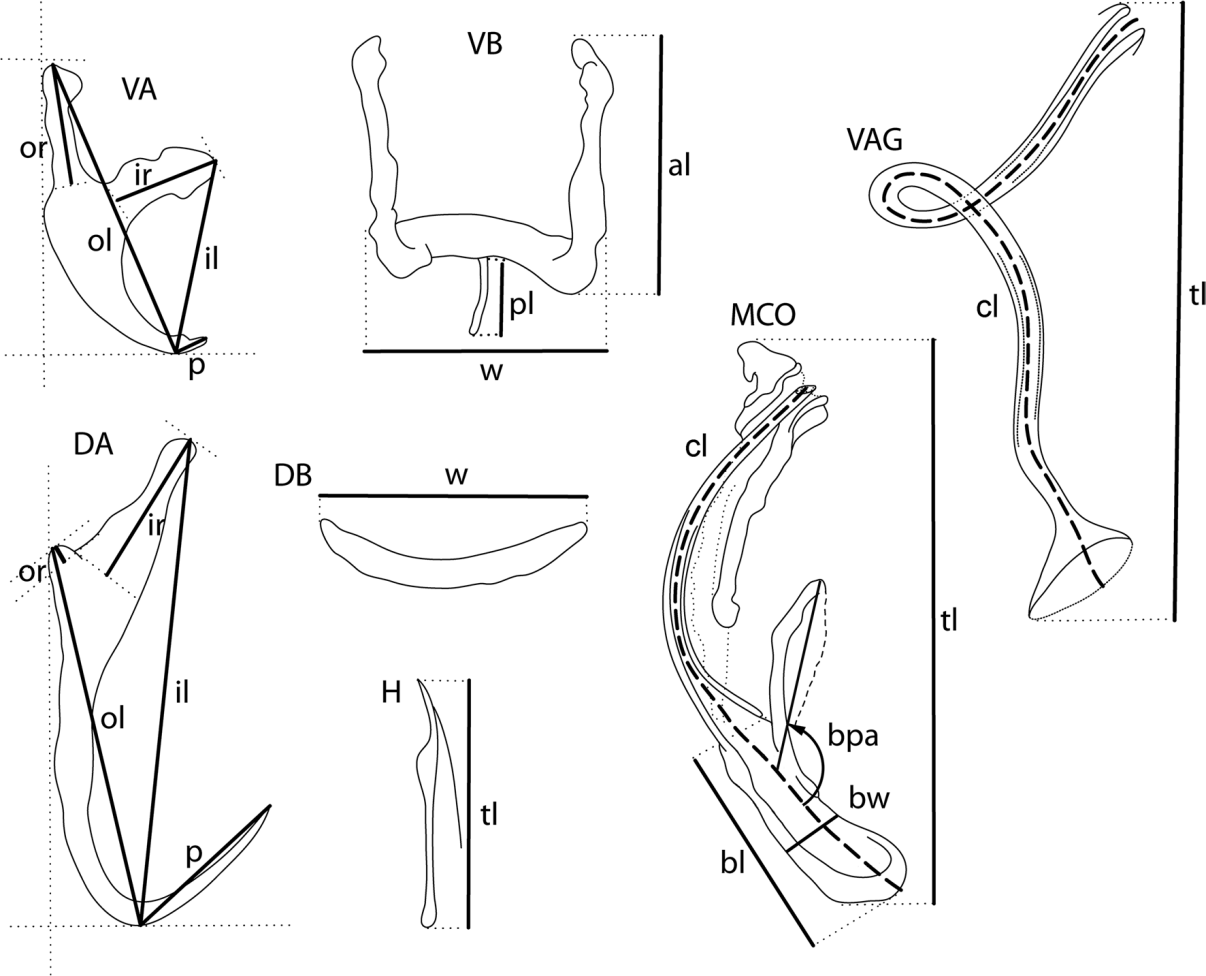
Scheme of measurements for sclerotized structures of *Characidotrema* spp. *Abbreviations*: VA, ventral anchor; DA, dorsal anchor; VB, ventral bar. DB, dorsal bar; H, hook. VAG, vagina; MCO, male copulatory organ; il, inner length; ol, outer length; ir, inner root length; or, outer root length; p, point length; tl, total length; w, width; al, arm lenght; pl, posteromedial projection length; cl, tube curved length; bl, base length; bw, base width; bpa, basal process angle

The mounted specimens (or their parts) were studied using an Olympus BX 61 microscope equipped with phase contrast optics. Drawings were made with the aid of a drawing attachment and edited with a graphic tablet compatible with Adobe Illustrator and Adobe Photoshop. Measurements, all in micrometres, were taken using digital image analysis (Stream Motion, version 1.9.2) and are given as the range followed by the mean and number (*n*) of specimens measured in parentheses. Schemes of measurements of the hard structures are provided in Fig. 1. Numbering of hook pairs (in Roman numerals I-VII) is that recommended by Mizelle [14]. Male copulatory organ is henceforth abbreviated to MCO.

After morphometric analysis, specimens (or their parts) fixed with GAP were remounted in Canada balsam according to Ergens [15] and deposited as type-material, morphological vouchers and hologenophores (see [16] for terminology). For comparative purposes, type-specimens of three previously described species of *Characidotrema* (syn. *Jainus*) deposited in the Royal Museum for Central Africa, Belgium (RMCA/MRAC), and one species deposited in the Institute of Parasitology, Czech Academy of Science, České Budějovice, Czech Republic (IPCAS), were examined. Type- and voucher specimens of monogeneans collected for the present study were deposited in the helminthological collection of the IPCAS (see below for the details).

### DNA extraction, amplification and sequencing

Total genomic DNA was separately extracted from each ethanol-fixed specimen (3–10 specimens per species) using a DNeasy® Blood & Tissue Kit (Qiagen, Hilden, Germany) following the manufacturerʼs instructions. Two fragments of nuclear ribosomal DNA, generally considered as suitable markers for monogenean species level determination [17], were used for molecular characterization: (i) a fragment spanning partial *18S* rDNA and internal transcribed spacer (*18S*-ITS1); and (ii) a fragment of partial *28S* rDNA (28S). Primer details and amplification conditions are given in Table 3. PCRs were carried out in a total volume of 30 μl containing 5 μl of DNA extract, 1× PCR buffer (Fermentas), 1.5 mM MgCl_2_, 200 µM of each dNTP, 0.5 μM (for *28S*) or 0.8 μM (for *18S*-ITS1) of each primer, and 1U of Taq polymerase (Fermentas). PCR amplicons were purified using an ExoSAP-IT™ (Affymetrix Inc., Santa Clara, USA) and sequenced directly from both strands using the PCR primers. DNA sequencing was carried out using BigDye® Terminator v3.1 Cycle Sequencing Kit (Applied Biosystems by Thermo Fisher Scientific, Prague, Czech Republic) and an Applied Biosystems 3130 Genetic Analyzer (Applied Biosystems). Sequences were assembled and edited using Sequencher software (Gene Codes Corp., Ann Arbor, MI, USA). Using BLASTn, the generated sequences were compared to the NCBI database in order to assess sequence similarity and to check for possible contamination. Newly obtained sequences of the *18S*-ITS1 and *28S* fragments from *Characidotrema* spp. were deposited in the GenBank database under the accession numbers MK014156-MK014161 and MK012538-MK012543. Hologenophores, i.e. vouchers from which molecular samples were directly derived (see [16] for terminology), were deposited in the helminthological collection of the IPCAS.

**Table 3:** Specification of primer pairs used for gene amplification and PCR conditions

Primer name	Marker	Sequence (5′–3′)	PCR cycling conditions	Reference
S1 (Forward)	*18S*-ITS1	ATT CCG ATA ACG AAC GAG ACT	94 °C for 2 min (39×: 94 °C for 60 s; 53 °C for 60 s; 72 °C for 90 s) 72 °C for 10 min	[44]
IR8 (Reverse)		GCT AGC TGC GTT CTT CAT CGA		[45]
C1 (Forward)	*28S*	ACC CGC TGA ATT TAA GCA T	94 °C for 2 min (39×: 94 °C for 20 s; 56 °C for 30 s; 72 °C for 90 s) 72 °C for 10 min	[46]
D2 (Reverse)		TGG TCC GTG TTT CAA GAC		[46]

### Genetic distances and phylogenetic reconstruction

Alignments of *18S*, ITS1 and *28S* were generated using MAFFT v.7 [18] and manually adjusted in BioEdit [19]. Interspecific genetic distances were determined using distance matrices (uncorrected p-distances) in MEGA 7 [20] separately for each genetic marker. To determine the position of *Characidotrema* spp. among other representatives of African dactylogyrid genera, phylogenetic analyses were conducted using Maximum Likelihood (ML) and Bayesian Inference (BI) methods. Phylogenetic reconstruction including 34 selected African dactylogyridean species (Table 4) was based on sequences of partial *28S* rDNA only. Three species belonging to the Anoplodiscidae and Diplectanidae were used as the outgroup. The *28S* sequences were aligned using MAFFT v. 7 [18] with the Q-INS-i algorithm [21] and other parameters set to default. Gaps and ambiguously aligned regions were removed from the alignment using GBlocks v. 0.91b [22] applying all options for a less stringent selection. ML analysis was conducted using the IQ-TREE [23] on the W-IQ-TREE web server [24]. The default “Auto” setting was selected to determine the best-fit substitution model. The TIM3+F+I+G4 model was selected as the optimal model of molecular evolution based on the Bayesian information criterion (BIC). Branch support was estimated using ultrafast bootstrap approximation [25] with 10,000 replicates. BI analysis was performed in MrBayes 3.2.1 [26]. However, since the selected substitution model is not implemented in MrBayes, the GTR+I+G was selected as the closest matching alternative. Four simultaneous chains (one cold and three heated) of the Markov Chain Monte Carlo (MCMC) algorithm were run twice for 10^7^ generations. Tree topologies were sampled every 100 generations, whereby the first 25% of trees from each run were discarded as “burn-in”. The remaining trees were used to construct majority-rule consensus trees and determine the Bayesian posterior probability (BPP) for each clade. The trees were visualized and edited in FigTree ver. 1.4.3. [27].

**Table 4:** Monogenean species used in the phylogenetic analyses, their hosts, geographical localities and GenBank numbers

Monogenean species	Host species	Locality	*28S* rDNA
Dactylogyridae Bychowsky, 1933			
*Characidotrema auritum* n. sp.	*Brycinus imberi*	South Africa, Zimbabwe	MK012538^a^
*Characidotrema brevipenis*	*Brycinus nurse*	Sudan	MK012539^a^
*Characidotrema nursei*	*Brycinus nurse*	Sudan	MK012540^a^
*Characidotrema pollex* n. sp.	*Brycinus nurse*	Sudan	MK012541^a^
*Characidotrema spinivaginus*	*Brycinus nurse*	Sudan	MK012542^a^
*Characidotrema vespertilio* n. sp.	*Brycinus imberi*	Cameroon, DR Congo	MK012543^a^
*Cichlidogyrus amphoratus*	*Coptodon guineensis*	Senegal	HE792772
*Cichlidogyrus casuarinus*	*Hemibates stenosoma*	Burundi	KX007819
*Cichlidogyrus cirratus*	*Oreochromis niloticus*	Senegal	HE792773
*Cichlidogyrus douellouae*	*Sarotherodon galilaeus*	Senegal	HE792774
*Cichlidogyrus njinei*	*Sarotherodon galilaeus*	Senegal	HE792775
*Cichlidogyrus tiberianus*	*Coptodon guineensis*	Senegal	HE792776
*Cichlidogyrus yanni*	*Coptodon guineensis*	Senegal	HE792777
*Dactylogyrus benhoussai*	*Carasobarbus moulouyensis*	Morocco	KX553862
*Dactylogyrus falsiphallus*	*Luciobarbus maghrebensis*	Morocco	KX553861
*Dactylogyrus scorpius*	*Luciobarbus rifensis*	Morocco	KX553860
*Dactylogyrus varius*	*Luciobarbus maghrebensis*	Morocco	KX553863
*Enterogyrus coronatus*	*Coptodon zillii*	Senegal	HQ010030
*Enterogyrus* sp. 1	*Sarotherodon galilaeus*	Senegal	HQ010032
*Enterogyrus* sp. 2	*Sarotherodon galilaeus*	Senegal	HQ010031
*Onchobdella aframae*	*Hemichromis fasciatus*	Senegal	HQ010033
*Onchobdella bopeleti*	*Hemichromis fasciatus*	Senegal	HQ010034
*Quadriacanthus bagrae*	*Bagrus docmak*	Sudan	KX685951
*Quadriacanthus clariadis*	*Clarias gariepinus*	Sudan	KX685952
*Quadriacanthus fornicatus*	*Clarias gariepinus*	Sudan	KX685953
*Quadriacanthus mandibulatus*	*Heterobranchus bidorsalis*	Sudan	KX685954
*Quadriacanthus pravus*	*Clarias gariepinus*	Sudan	KX685955
*Quadriacanthus zuheiri*	*Clarias gariepinus*	Sudan	KX685956
*Scutogyrus bailloni*	*Sarotherodon galilaeus*	Ivory Coast	HE792778
*Scutogyrus longicornis*	*Oreochromis niloticus*	Senegal	HQ010035
*Scutogyrus minus*	*Sarotherodon melanotheron*	Ivory Coast	HE792779
*Schilbetrema* sp.	*Pareutropius debauwi*	West Africa	KP056244
*Schilbetrema* sp.	*Pareutropius debauwi*	West Africa	KP056243
*Synodontella zambezensis*	*Synodontis zambezensis*	South Africa	LT220022
Diplectanidae Monticelli, 1903			
*Diplectanum blaiense*	*Sillago sihama*	China	AY553627
*Laticola paralatesi*	*Lates calcarifer*	Australia	KP313568
Anoplodiscidae Tagliani, 1912			
*Anoplodiscus cirrusspiralis*	*Sparus aurata*	Australia	AF382060

^a^Sequence generated in this study

*Note*: Species of the Anoplodiscidae and Diplectanidae were used as outgroups

## Results

**Family Dactylogyridae Bychowsky, 1933**

**Genus** ***Characidotrema*** **Paperna & Thurston, 1968**

***Characidotrema brevipenis*** **Paperna, 1969**

Syn. *Jainus brevipenis* (Paperna, 1969) Paperna, 1979 [3]

***Type-host***: *Brycinus nurse* (Rüppell) (syn. *Alestes nurse*).

***Type-locality***: Lake Volta, Ghana.

***Other records***: *Alestes baremoze*, Lake Volta, Ghana [7, 8]; *Brycinus* cf. *nurse*, River Mono, Togo [3].

***Present material***: Ex *Brycinus nurse*, River Gambia, Simenti (13°01′23.40″N, 13°17′21.00″W), Mare de Simenti Oxbow (13°01′47.39″N, 13°17′35.99″W), Senegal; River White Nile, Kosti (13°10′18.58″N, 32°40′19.24″E), Sudan.

***Site on host***: Gill lamellae.

***Type-specimens examined***: *Characidotrema brevipenis* Paperna, 1969 (MRAC 35.913; holotype, paratypes).

***Voucher material***: Six voucher specimens including a hologenophore in IPCAS (M-686).

***Representative DNA sequences***: GenBank: MK012539 (*28S* rDNA) and MK014157 (*18S*-ITS1 rDNA).

### Measurements

**Fig. 2:**
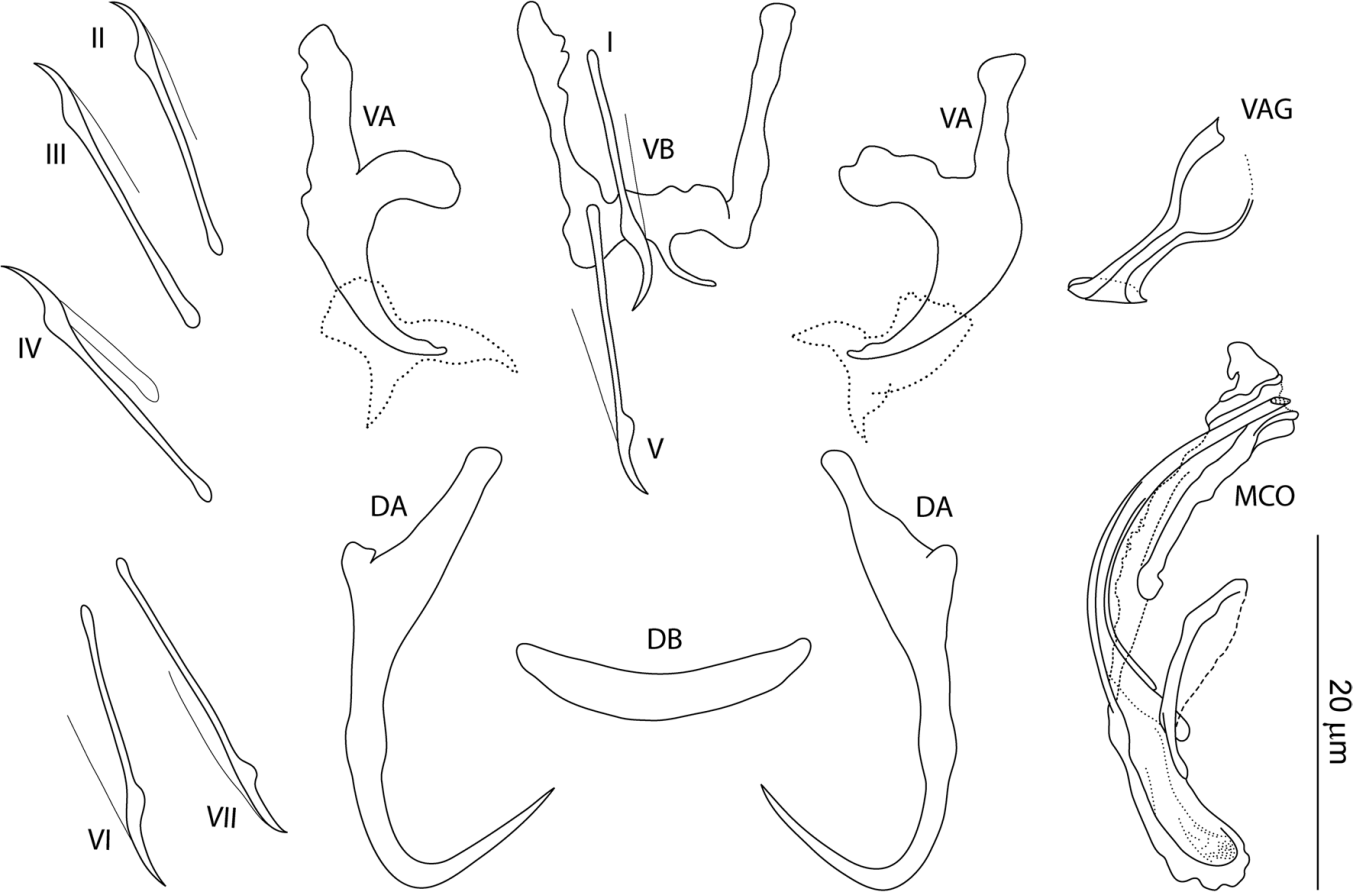
*Characidotrema brevipenis* Paperna, 1969. Sclerotized structures. *Abbreviations*: VA, ventral anchor; DA, dorsal anchor; VB, ventral bar; DB, dorsal bar; I-VII, hooks; VAG, vagina; MCO, male copulatory organ

[Based on 10 specimens fixed in GAP; Fig. 2]. Ventral anchor: inner length 12–13 (13; *n *= 10); outer length 20–21 (20; *n* = 10); inner root length 6–7 (7; *n* = 10); outer root length 7 (*n* = 10); point length 2–3 (3; *n* = 10). Dorsal anchor: inner length 24–25 (24; *n* = 10); outer length 19–20 (20; *n* = 10); inner root length 7–9 (7; *n* = 10); outer root length 1 (*n* = 10); point length 8–10 (9; *n* = 10). Ventral bar: width 11–16 (12; *n* = 10); arm length 12–14 (13; *n* = 10); posteromedial projection length 4–5 (4; *n* = 10). Dorsal bar: width 17–19 (18; *n* = 10). Hooks, pairs I-VII: total length 15–18 (17; *n* = 10). Vagina sclerotized: straight length 13–18 (17; *n* = 10). MCO: total straight length 32–34 (33; *n* = 10); tube curved length 38–41 (39; *n* = 10); base length 13–14 (14; *n* = 10); base width 3–5 (4; *n* = 10); finger-like basal process moderately developed (slightly shorter than the base), rising from the base at an angle of about 100°.

### Molecular characterisation

The sequence of the *18S*-ITS1 region of *C. brevipenis* was 978 bp long, of which 483 bp corresponded to the partial *18S* rDNA and 495 bp corresponded to the ITS1 region. The nucleotide sequence of the partial *28S* region was 761 bp long. Four specimens of *C. brevipenis* from Sudan (White and Blue Nile) were sequenced; no intraspecific sequence variation was found.

### Differential diagnosis

On the basis of the comparative morphology of the sclerotized structures, our specimens are conspecific with the type-specimens of *C. brevipenis* (MRAC M. T. 35.913 A) collected from *Brycinus nurse* in Ghana by Paperna [7]. Measurements of the haptoral and copulatory sclerites of the present specimens fall within the ranges provided by Kritsky et al. [3] in their redescription of *C. brevipenis* from *B.* cf. *nurse* in Togo. *Characidotrema brevipenis* most closely resembles *C. undifera* Kritsky, Kulo & Boeger, 1987 and *C. zelotes* Kritsky, Kulo & Boeger, 1987 in the general morphology of the haptoral sclerites and the MCO [3]. It differs from these two species by having a longer (39 *vs* 34 µm in *C. undifera*; 39 *vs* 28–29 µm in *C. zelotes*) and more smoothly curved copulatory tube (a tube with a tighter curve and recurved termination is present in *C. undifera* and *C. zelotes*). Kritsky et al. [3] recognized also the morphological similarity between *C. brevipenis* and *C. nzoiae* (Paperna, 1979), stating that *C. brevipenis* differs from the latter species by having a more elongate base of the copulatory tube. Nevertheless, the most apparent feature that distinguishes *C. brevipenis* from all the above-mentioned congeners is the goblet-shaped vagina.

***Characidotrema nursei*** **Ergens, 1973**

Syns. *Jainus longipenis* Paperna, 1973; *Jainus nursei* (Ergens, 1973) Paperna, 1979 [3]

***Type-host***: *Brycinus nurse* (syn. *Alestes nurse*).

***Type-locality***: River Nile, Cairo, Egypt.

***Other records***: *Brycinus nurse* (syn. *Alestes nurse*), Lake Albert, Uganda [4, 8].

***Present material***: Ex *Brycinus nurse*, River Blue Nile, Sennar (13°32′31.09″N, 33°37′15.79″E), River White Nile, Kosti (13°10′18.58″N, 32°40′19.24″E), Sudan.

***Site on host***: Gill lamellae.

***Type-specimens examined***: *Characidotrema nursei* Ergens, 1973 (IPCAS M-282; holotype); *Jainus longipenis* Paperna, 1973 (MRAC 35.918 IIII; holotype).

***Voucher material***: Six voucher specimens including a hologenophore in IPCAS (M-282).

***Representative DNA sequences***: GenBank: MK012540 (*28S* rDNA) and MK014158 (*18S*-ITS1 rDNA).

### Measurements

**Fig. 3:**
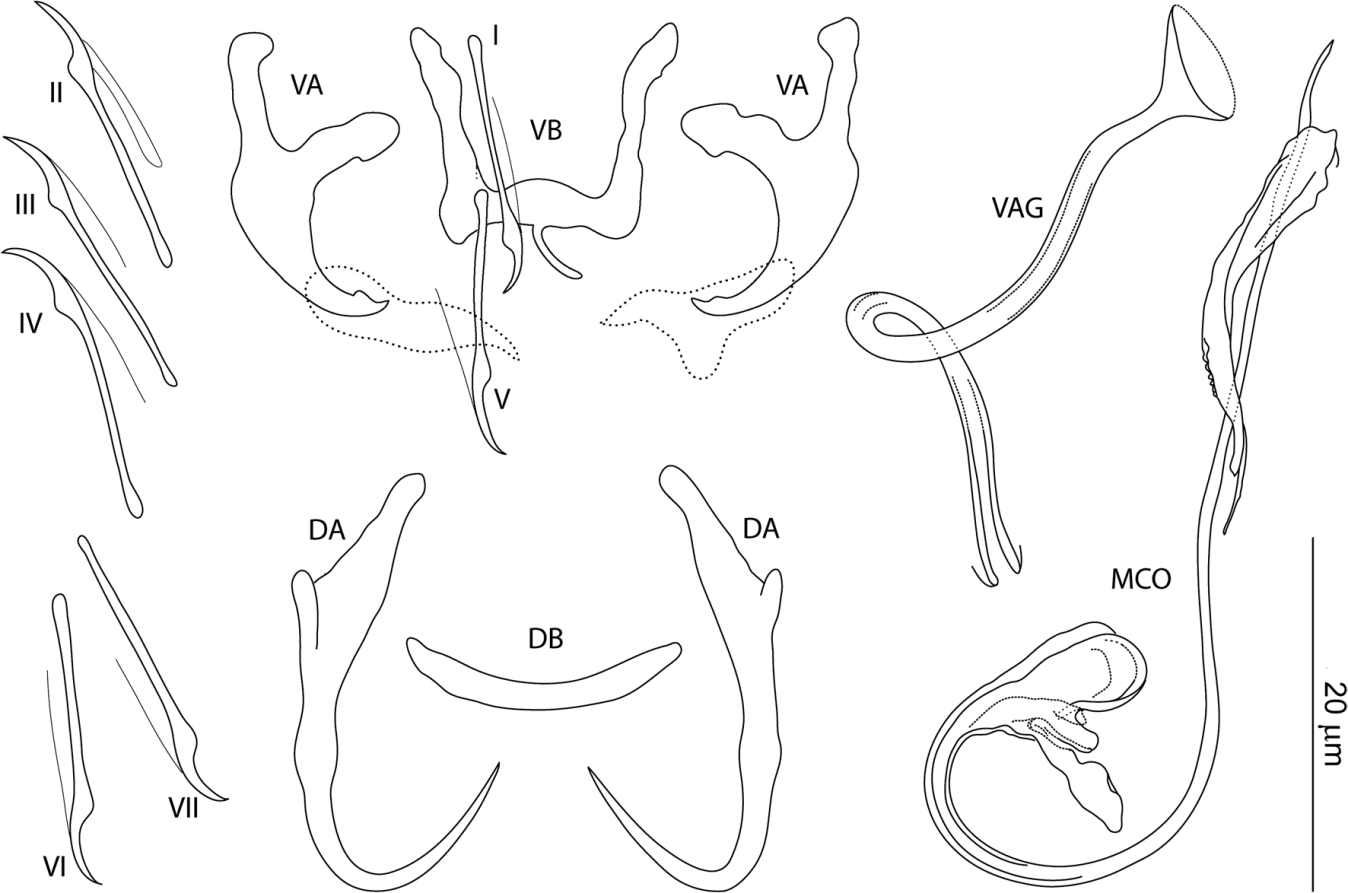
*Characidotrema nursei* Ergens, 1973. Sclerotized structures. *Abbreviations*: VA, ventral anchor; DA, dorsal anchor; VB, ventral bar; DB, dorsal bar; I-VII, hooks; VAG, vagina; MCO, male copulatory organ

[Based on 10 specimens fixed in GAP; Fig. 3]. Ventral anchor: inner length 10–12 (11; *n* = 10); outer length 17–19 (19; *n* = 10); inner root length 5–6 (6; *n* = 10); outer root length 7–8 (7; *n* = 10); point length 2–3 (2; *n* = 10). Dorsal anchor: inner length 23–25 (24; *n* = 10); outer length 17–19 (18; *n* = 10); inner root length 8–9 (9; *n* = 10); outer root length 1 (*n* = 10); point length 9–11 (10; *n* = 10). Ventral bar: width 10–18 (14; *n* = 10); arm length 8–14 (11; *n* = 10); posteromedial projection length 4–5 (4; *n* = 7). Dorsal bar: width 15–17 (16; *n* = 10). Hooks, pairs I-VII: total length 15–18 (16; *n* = 10). Vagina sclerotized: straight length 31–44 (36; *n* = 10); curved length 49–54 (51; *n* = 10). MCO: total straight length 34–50 (43; *n* = 10); tube curved length 70–75 (72; *n* = 10); base length 8–10 (8; *n* = 10); base width 4–6 (5; *n* = 10); finger-like basal process well developed (about the same length as the base), rising from the base at an angle of about 70°.

### Molecular characterisation

The combined *18S*-ITS1 sequence of *C. nursei* was 979 bp long, of which 483 bp corresponded to the *18S* rDNA and 496 bp corresponded to the ITS1 region. The sequence of partial *28S* rDNA was 836 bp long. Ten specimens from Sudan (Blue Nile) were sequenced and no intraspecific variability was found.

### Differential diagnosis

This species was originally described from the gills of *Brycinus nurse* in Egypt by Ergens [9]. Later, but in the same year, Paperna [4] proposed *Jainus longipenis* from the same host in Uganda. Under the revision of *Characidotrema*, Kritsky et al. [3] considered *Jainus longipenis* and *Jainus* cf. *longipenis* of Paperna [8] junior subjective synonyms of *C. nursei* based on the comparative morphology of the sclerotized structures in the type- and voucher specimens of all three forms. At the same time, however, they pointed out that the sclerotized structures (especially the MCO) of the voucher of *Jainus* cf. *longipenis* (MRAC 35.907) reported on *Brycinus leuciscus* (syn. *Alestes leuciscus*) in Ghana were somewhat smaller (MCO length 50 µm) than those of the type-specimens of *C. nursei* and *J. longipenis* (MCO length 68–71 µm). Furthermore, in their figures (figures 20–32 in [3]), the MCO of *J.* cf. *longipenis* differs from that of *C. nursei* (syn. *J. longipenis*) by having a markedly smaller base and a simple finger-like basal process arising from the distal part of the base (*vs* complex, divided into two parts). Since the morphology of the MCO (especially the structure of the base) is the most important feature distinguishing species in the genus, we consider that *C.* cf. *longipenis* most probably represents an undescribed species of *Characidotrema*. As a result, we hesitate to list *B. leuciscus* as a host for *C. nursei* (see the taxonomic summary for host and locality details). The record of *C. nursei* on *Alestes dentex* in Egypt [10] is probably erroneous. Although the haptoral sclerites show some similarities to those of *C. nursei*, the size and shape of the MCO reported by Molnár & Mossalam [10] suggest that these authors were dealing with a different species. In their paper, the MCO is depicted as J-shaped with a comparatively shorter copulatory tube than that in *C. nursei* (40–48 *vs* 70 µm in the holotype of *C. nursei*). The general shape and size of the MCO as well as the vagina, while comparatively more tangled (see figure 1 in [10]), correspond rather to those in the original description of *C. ruahae* (Paperna, 1979) Kritsky, Kulo & Boeger, 1987 (syn. *Jainus brevipenis ruahae*; [8]). However, a formal proposal of synonymy between *C. nursei* of Molnár & Mossalam [10] and *C. ruahae* will require examination of new material from *A. dentex* from the type-locality in Egypt, as these authors apparently did not deposit specimens of their species in any parasitological collection. Comparisons of the present specimens with the type-specimens of *C. nursei* from Egypt and *J. longipenis* from Uganda confirm their conspecifity. Measurements of the sclerites of the holotypes fall within ranges reported herein for specimens collected from Sudan. In addition, they all share a morphologically identical MCO characterized by finger-like basal process divided into two parts (longer part with irregular margins). Thus, the finding of *C. nursei* in Sudan represents a new geographical record for this species.

***Characidotrema spinivaginus*** **(Paperna, 1973) Kritsky, Kulo & Boeger, 1987**

Syn. *Jainus spinivaginus* Paperna, 1973 [3]

***Type-host***: *Brycinus nurse* (syn. *Alestes nurse*).

***Type-locality***: Lake Albert, Uganda.

***Other record***: *Brycinus nurse* (syn. *Alestes nurse*), Lake Volta, Ghana [8].

***Present material***: Ex *Brycinus nurse*, River Gambia, Simenti (13°01′23.40″N, 13°17′21.00″W), Senegal; River Blue Nile, Sennar (13°32′31.09″N, 33°37′15.79E), River White Nile, Kosti (13°10′18.58″N, 32°40′19.24″E), Sudan.

***Site on host***: Gill lamellae.

***Type-specimens examined***: *Jainus spinivaginus* Paperna, 1973 (MRAC 35.942; holotype).

***Voucher material***: Six voucher specimens including a hologenophore in IPCAS (M-690).

***Representative DNA sequences***: GenBank: MK012542 (*28S* rDNA) and MK014160 (*18S*-ITS1 rDNA).

### Description

**Fig. 4:**
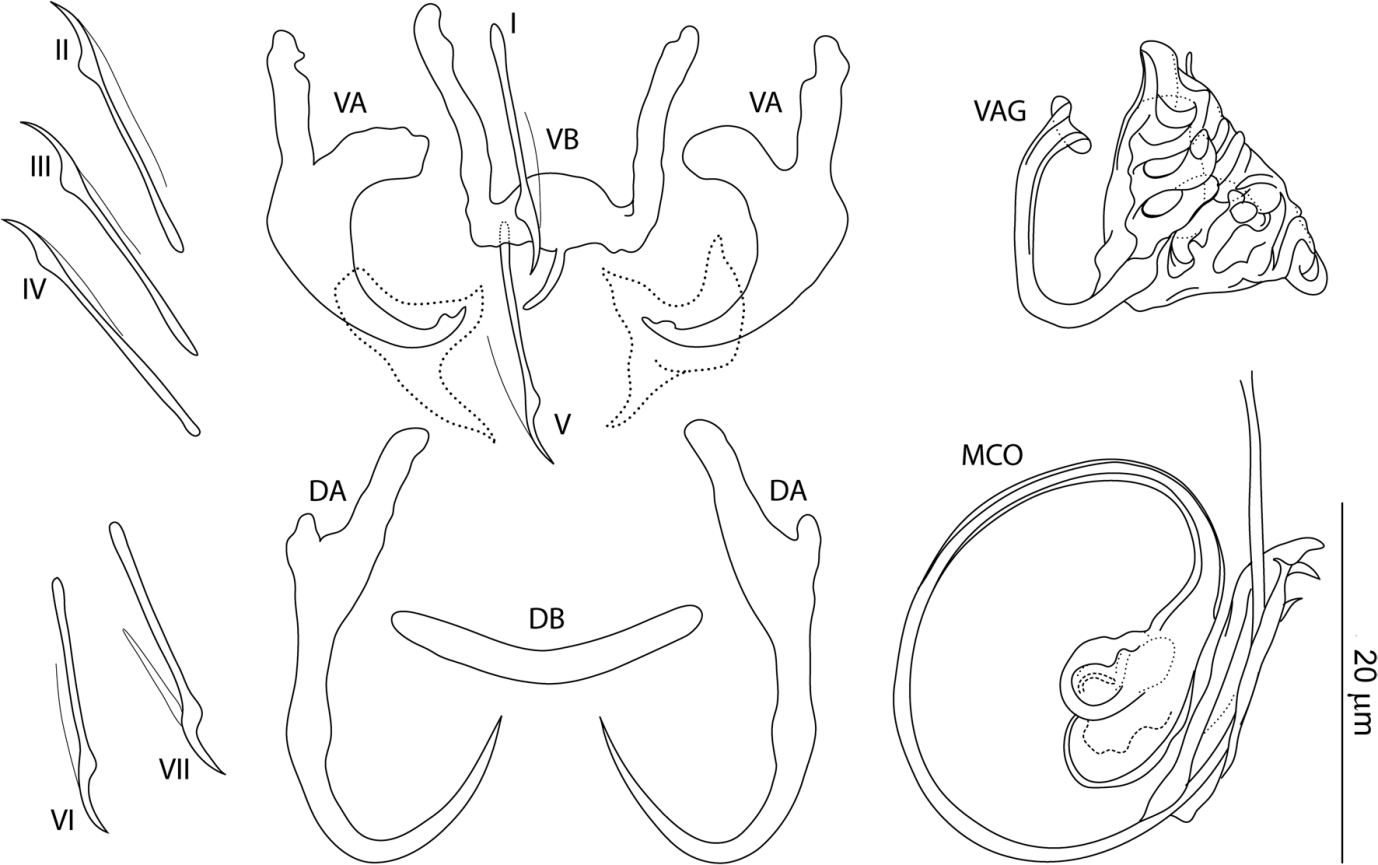
*Characidotrema spinivaginus* (Paperna, 1973) Kritsky, Kulo & Boeger, 1987. Sclerotized structures. *Abbreviations*: VA, ventral anchor; DA, dorsal anchor; VB, ventral bar; DB, dorsal bar; I-VII, hooks; VAG, vagina; MCO, male copulatory organ

[Based on 10 specimens fixed in GAP; Fig. 4]. Ventral anchors with roots nearly perpendicular to each other, evenly arced (recurved) inner root, elongate outer root, poorly differentiated base and shaft, and short scoop-shaped point; anchor filaments poorly differentiated; supporting sheath-like structure usually observed; inner length 12–13 (13; *n* = 10); outer length 19–22 (21; *n* = 10); inner root length 6–7 (6; *n* = 10); outer root length 8–9 (9; *n* = 10); point length 3–4 (4; *n* = 10). Dorsal anchors with elongate inner root, short outer root, elongate slightly curved shaft with submedial bump-like swelling, and long evenly curved point; anchor filaments often visible; inner length 23–24 (24; *n* = 10); outer length 19–20 (20; *n* = 10); inner root length 7–9 (8; *n* = 10); outer root length 2–3 (2; *n* = 10); point length 9–10 (10; *n* = 10). Ventral bar 11–14 (13; *n* = 10) wide; anterior arms 12–18 (15; *n* = 10) long; posteromedial projection 4–6 (5; *n* = 10) long. Dorsal bar broadly V-shaped, 16–18 (17; *n* = 10) wide. Hooks similar; each with undilated shank, poorly developed thumb, 14–16 (15; *n* = 10) long; FH loop 0.5 shank length. Vagina sclerotized, a triangular structure armed with spines; curved length 25–39 (31; *n* = 10). MCO comprising copulatory tube, accessory piece; total straight length 25–39 (31; *n* = 10). Copulatory tube a coil of about one ring; finger-like basal process short (about 2/3 length of the base), rising from the base at an angle of about 30°; tube curved length 77–89 (82; *n* = 10); base length 9–13 (12; *n* = 10); base width 4–6 (5; *n* = 10). Accessory piece variable, guiding distal part of the copulatory tube.

### Molecular characterisation

The combined *18S*-ITS1 sequence of *C. spinivaginus* was 942 bp long, of which 483 bp corresponded to the *18S* rDNA and 459 bp corresponded to the ITS1 region. The sequence of partial *28S* rDNA was 762 bp long. Two specimens were sequenced from Sudan (White Nile: Kosti), and no intraspecific variability was observed.

### Differential diagnosis

This species was incompletely described (without the drawings of haptoral structures) by Paperna [4, 8] as *Jainus spinivaginus* from *B. nurse* in Ghana and Uganda. Kritsky et al. [3] subsequently transferred the species to *Characidotrema* and supplemented the original description with drawings of both the ventral and dorsal anchor-bar complexes based on a re-examination of the holotype. However, they did not provide drawings of the hooks, probably because of the poor condition of the holotype. Morphological comparisons of the present specimens with the holotype of *C. spinivaginus* (MRAC M. T. 35.942) confirmed that all are conspecific. Also, the measurements of the sclerotized structures of the present specimens do not appear to vary significantly from those originally reported by Paperna [4, 8] since only two specimens were originally measured. Thus, the present findings of *C. spinivaginus* on the gills of *B. nurse* (Senegal, Sudan) represent new host and geographical records for this species. In addition, the taxonomically important sclerotized structures are illustrated here in one figure for the first time and the following information from the present specimens supplements the original description: (i) the accessory piece guiding the distal portion of the copulatory tube is present (not mentioned/depicted in the original description); (ii) the finger-like basal process, while clearly illustrated in the original drawing of the MCO, was mistakenly considered as an accessory piece by Paperna [8]; and (iii) delicate hooks are characterized by an undilated shank and poorly developed thumb (not described by Paperna [8]).

***Characidotrema zelotes*** **Kritsky, Kulo & Boeger, 1987**

***Type-host***: *Brycinus* cf. *nurse* (syn. *Alestes* cf. *nurse*).

***Type-locality***: River Mono, Togo.

***Present material***: Ex *Brycinus nurse*, River Gambia, Simenti (13°01′23.40″N, 13°17′21.00″W), Senegal; River White Nile, Kosti (13°10′18.58″N, 32°40′19.24″E), Sudan.

***Site on host***: Gill lamellae.

***Voucher material***: Five voucher specimens in IPCAS (M-691).

### Measurements

**Fig. 5:**
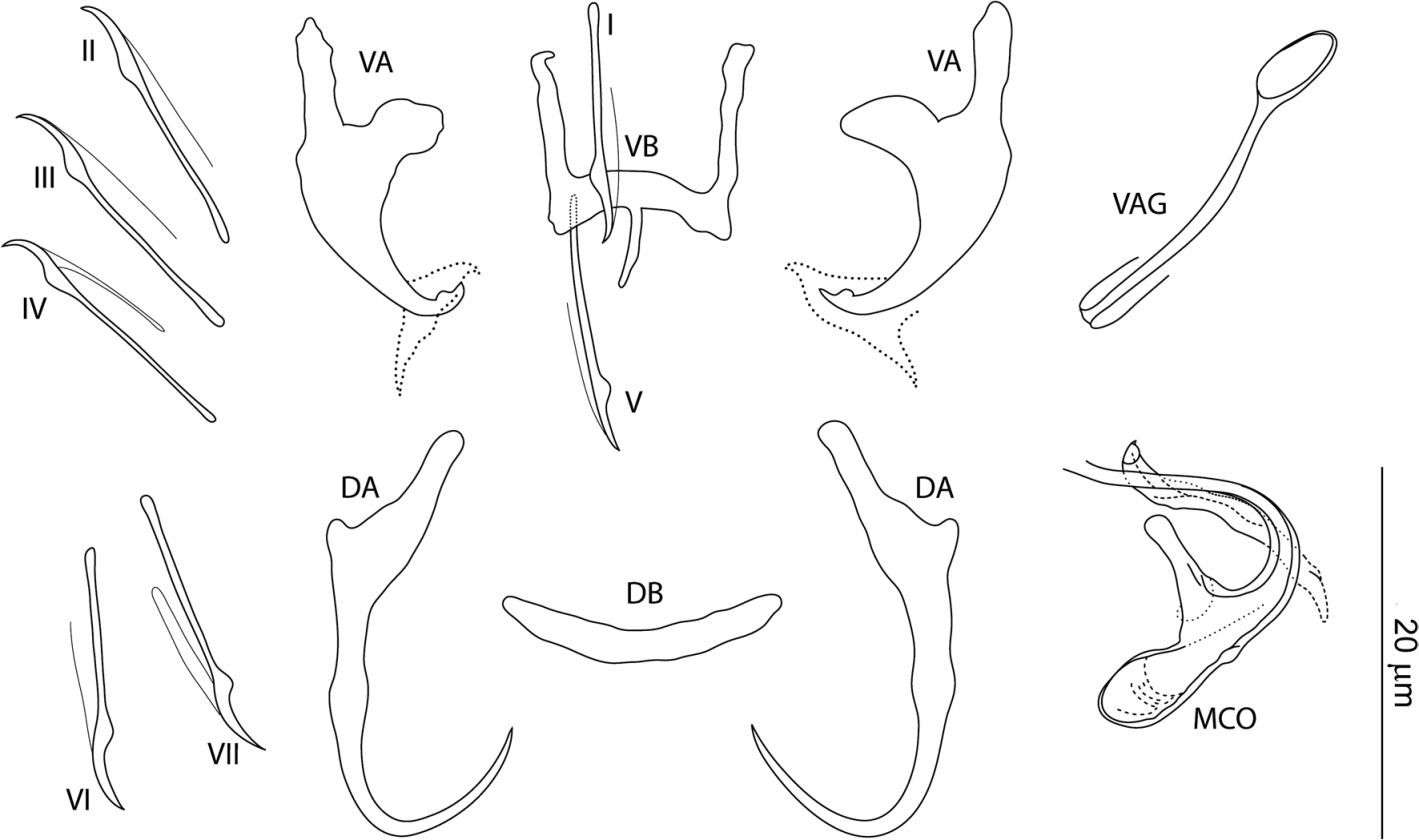
*Characidotrema zelotes* Kritsky, Kulo & Boeger, 1987. Sclerotized structures. *Abbreviations*: VA, ventral anchor; DA, dorsal anchor; VB, ventral bar; DB, dorsal bar; I-VII, hooks; VAG, vagina; MCO, male copulatory organ

[Based on 6 specimens fixed in GAP; Fig. 5]: Ventral anchor: inner length 10–11 (11; *n* = 6); outer length 17–18 (18; *n* = 6); inner root length 5–7 (6; *n* = 6); outer root length 7–8 (8; *n* = 6); point length 3 (*n* = 6). Dorsal anchor: inner length 23–24 (23; *n* = 6); outer length 18 (*n* = 6); inner root length 8–9 (8; *n* = 6); outer root length 1–2 (1; *n* = 6); point length 9–11 (10; *n* = 6). Ventral bar: width 11–12 (11; *n* = 6); arm length 2 (*n* = 6); posteromedial projection length 3–6 (4; *n* = 6). Dorsal bar: width 15–17 (16; *n* = 6). Hooks, pairs I -VII: total length 14–15 (15; *n* = 10). Vagina (weakly sclerotized): straight length 21–23 (22; *n* = 6); curved length 22–25 (23; *n* = 6). MCO: total straight length 16–17 (16; *n* = 6); tube curved length 27–30 (29; *n* = 6); base length 6–10 (9; *n* = 6); base width 3–4 (3; *n* = 6); finger-like basal process well developed (about the same length as the base), rising from the base at an angle of about 90°.

### Differential diagnosis

The sclerotized structures of the present specimens correspond both in size and morphology to those in the original description of *C. zelotes* by Kritsky et al. [3]. *Characidotrema zelotes* is most similar to *C. undifera* Kritsky, Kulo & Boeger, 1987 from *Brycinus* cf. *nurse* collected in Togo [3], but it differs from the latter species by possessing smaller haptoral sclerites and by lacking a subterminal angular bend of the copulatory tube. The findings of *C. zelotes* in Senegal and Sudan represent new locality records for this species.

***Characidotrema pollex*** **Kičinjaová & Řehulková n. sp.**

***Type-host***: *Brycinus nurse* (Rüppell, 1832).

***Type-locality***: River Blue Nile, Sennar (13°32′31.09″N, 33°37′15.79″E), Sudan.

***Other localities***: River Gambia, Simenti (13°01′23.40″N, 13°17′21.00″W), Senegal; River White Nile, Kosti (13°10′18.58″N, 32°40′19.24″E), Sudan.

***Site on host***: Gill lamellae.

***Type-material***: Holotype, nine paratypes and a hologenophore in IPCAS (M-688).

***Representative DNA sequences***: GenBank: MK012541 (*28S* rDNA) and MK014159 (*18S*-ITS1 rDNA).

***ZooBank registration***: To comply with the regulations set out in article 8.5 of the amended 2012 version of the International Code of Zoological Nomenclature (ICZN) [28], details of the new species have been submitted to ZooBank. The Life Science Identifier (LSID) of the article is urn:lsid:zoobank.org:pub:506A57E6-A1AF-4978-B72D-1C174AAB6F70. The LSID for the new name *Characidotrema pollex* Kičinjaová & Řehulková n. sp. is urn:lsid:zoobank.org:act:8847563E-A284-46BB-A839-C173D4EDE563.

***Etymology:*** The specific epithet (a noun) is from the Latin (*pollex* = thumb) and refers to the thumb-like process arising from the base of the copulatory tube.

### Description

**Fig. 6:**
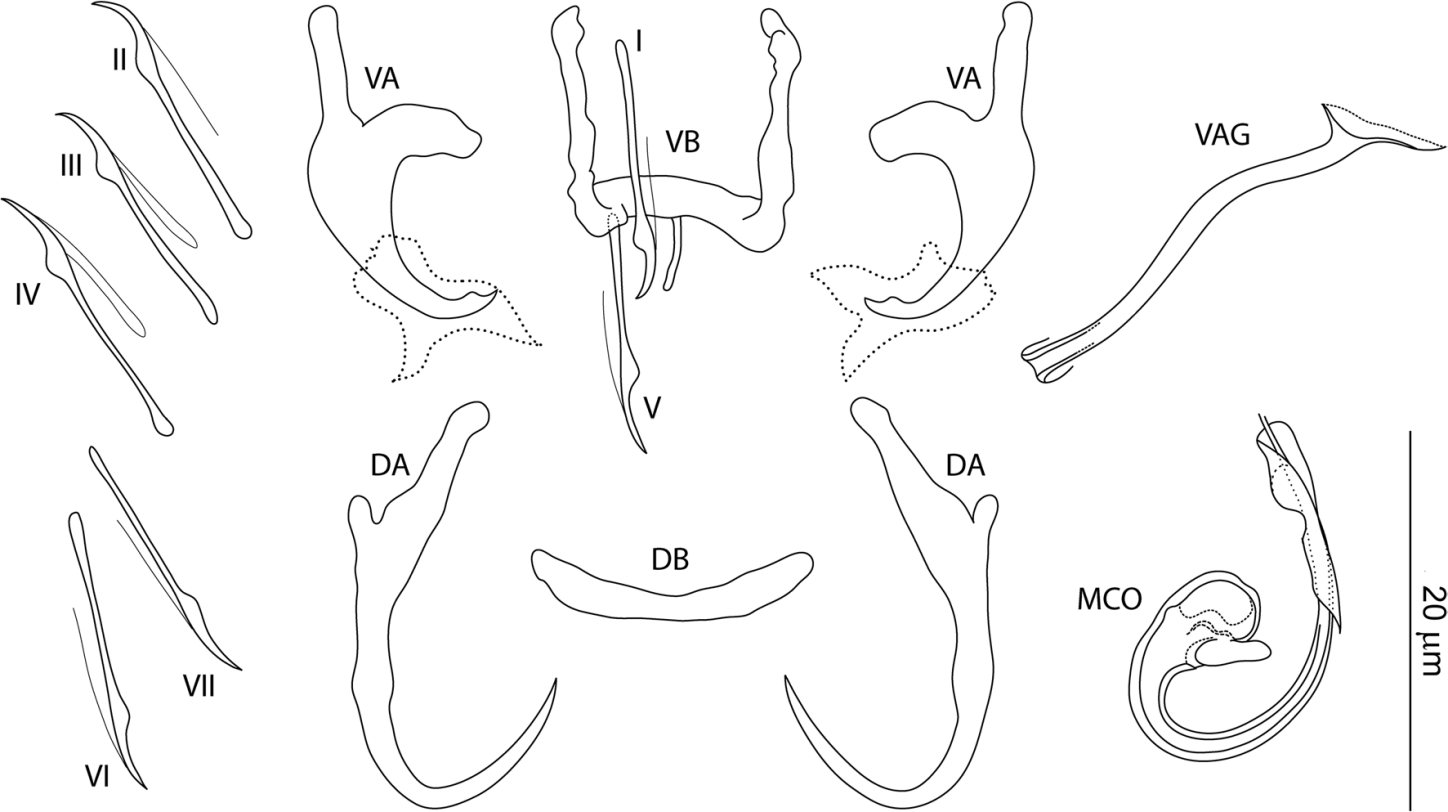
*Characidotrema pollex* Kičinjaová & Řehulková n. sp. Sclerotized structures. *Abbreviations*: VA, ventral anchor; DA, dorsal anchor; VB, ventral bar; DB, dorsal bar; I-VII, hooks; VAG, vagina; MCO, male copulatory organ

[Based on 10 specimens fixed in GAP; Fig. 6]. Ventral anchors with roots perpendicular to each other, evenly arced (recurved) inner root, elongate outer root, poorly differentiated base and shaft, and short scoop-shaped point; anchor filaments poorly differentiated; supporting sheath-like structure usually observed; inner length 9–11 (11; *n* = 10); outer length 18–20 (19; *n* = 10); inner root length 5–6 (5; *n* = 10); outer root length 6–8 (7; *n* = 10); point length 2–3 (3; *n* = 10). Dorsal anchors with elongate inner root, short outer root, elongate slightly curved shaft with submedial bump-like swelling, and elongate slightly recurved point; anchor filaments often visible; inner length 21–22 (21; *n* = 10); outer length 15–19 (17; *n* = 10); inner root length 6–8 (7; *n* = 10); outer root length 2–3 (2; *n* = 10); point length 8–10 (9; *n* = 10). Ventral bar 11–15 (13; *n* = 10) wide; anterior arms 10–14 (12; *n* = 10) long; posteromedial projection 3–4 (3; *n* = 10) long. Dorsal bar broadly V-shaped, 15–17 (16; *n* = 10) wide. Hooks similar; each with undilated shank, poorly developed thumb, 12–15 (14; *n* = 10) long. Vagina sclerotized, a slightly wavy tube; straight length 19–26 (22; *n* = 10); curved length 22–29 (26; *n* = 5). MCO comprising copulatory tube and accessory piece; total straight length 14–19 (17; *n* = 10). Copulatory tube J-shaped; base short, rounded; finger-like basal process about same length as base, rising from base at an angle of about 40°; tube curved length 31–39 (36; *n* = 10); base length 5–7 (6; *n* = 10); base width 3–5 (5; *n* = 10). Accessory piece a sheath guiding distal part of copulatory tube.

### Molecular characterisation

The combined *18S*-ITS1 sequence of *C. pollex* was 882 bp long, of which 483 bp corresponded to the *18S* rDNA and 399 bp corresponded to the ITS1 region. The sequence of partial *28S* rDNA was 829 bp long. Three specimens were sequenced from Sudan (White and Blue Nile) and no intraspecies variability for *28S* rDNA *18S*-ITS1 was observed.

### Differential diagnosis

*Characidotrema pollex* n. sp. belongs to the group of congeners having a copulatory tube with a finger-like basal process that is not articulated to the accessory piece: *C. brevipenis* Paperna, 1969; *C. nursei* Ergens, 1973; *C. nzoiae* (Paperna, 1979); *C. regia* Birgi, 1988 (doubtful due to poor original drawing); *C. ruahae* (Paperna, 1979); *C. spinivaginus* (Paperna, 1973); *C. undifera* Kritsky, Kulo & Boeger, 1987; and *C. zelotes* Kritsky, Kulo & Boeger, 1987. The size and shape of the process, and the angle at which it rises from the base represent important features for differentiating between these species. *Characidotrema pollex* n. sp. differs from all the above congeners by having J-shaped copulatory tube with shortened base and relatively small finger-like process, which rises from the base at an angle of about 40°. It most resembles *C. ruahae*, from which it differs by the latter species possessing a comparatively longer finger-like process, rising from the base at an angle of about 90°.

*Characidotrema pollex* n. sp. is morphologically similar to *Jainus* cf. *longipenis* of Paperna (1979) reported on *Brycinus leuciscus* (syn. *Alestes leuciscus*) in Ghana. Although the voucher specimen of this form was not examined, illustrations of the haptoral structures and MCO were provided by Kritsky et al. [3] (see also remarks to *C. nursei*). On the basis of their drawings, both species have a copulatory tube with a short finger-like basal process rising from a small base at an angle of about 40°. The new species differs from *J.* cf. *longipenis* by having a comparatively shorter (31–39 *vs* 50 µm) and J-shaped (*vs* coiled) shaft of the tube. However, these morphological differences are relatively small, and in absence of comparative material, new specimens of *J*. cf. *longipenis* will be needed to make a redescription and to obtain molecular data. Then a decision on synonymy of *C. polex* n. sp. with *J.* cf. *longipenis* may be made.

***Characidotrema auritum*** **Kičinjaová & Řehulková n. sp.**

***Type-host***: *Brycinus imberi* (Peters, 1852).

***Type-locality***: River Pongola (26°52′57.71″S, 32°18′40.68″E), South Africa.

***Other locality***: Lake Kariba (16°04′50.99″S, 28°52′04.00″E), Zimbabwe.

***Site on host***: Gill lamellae.

***Type-material***: Holotype, nine paratypes and a hologenophore in IPCAS (M-687).

***Representative DNA sequences***: GenBank: MK012538 (*28S* rDNA) and MK014156 (*18S*-ITS1 rDNA).

***ZooBank registration***: To comply with the regulations set out in article 8.5 of the amended 2012 version of the International Code of Zoological Nomenclature (ICZN) [28], details of the new species have been submitted to ZooBank. The Life Science Identifier (LSID) of the article is urn:lsid:zoobank.org:pub:506A57E6-A1AF-4978-B72D-1C174AAB6F70. The LSID for the new name *Characidotrema auritum* Kičinjaová & Řehulková n. sp. is urn:lsid:zoobank.org:act:BDAB59B9-0D15-4FA4-AD27-5B4D408A2AEB.

***Etymology***: The specific epithet (an adjective) is from the Latin (*auritum* = having the form of an ear) and refers to the ear-like terminal branches of the accessory piece.

### Description

**Fig. 7:**
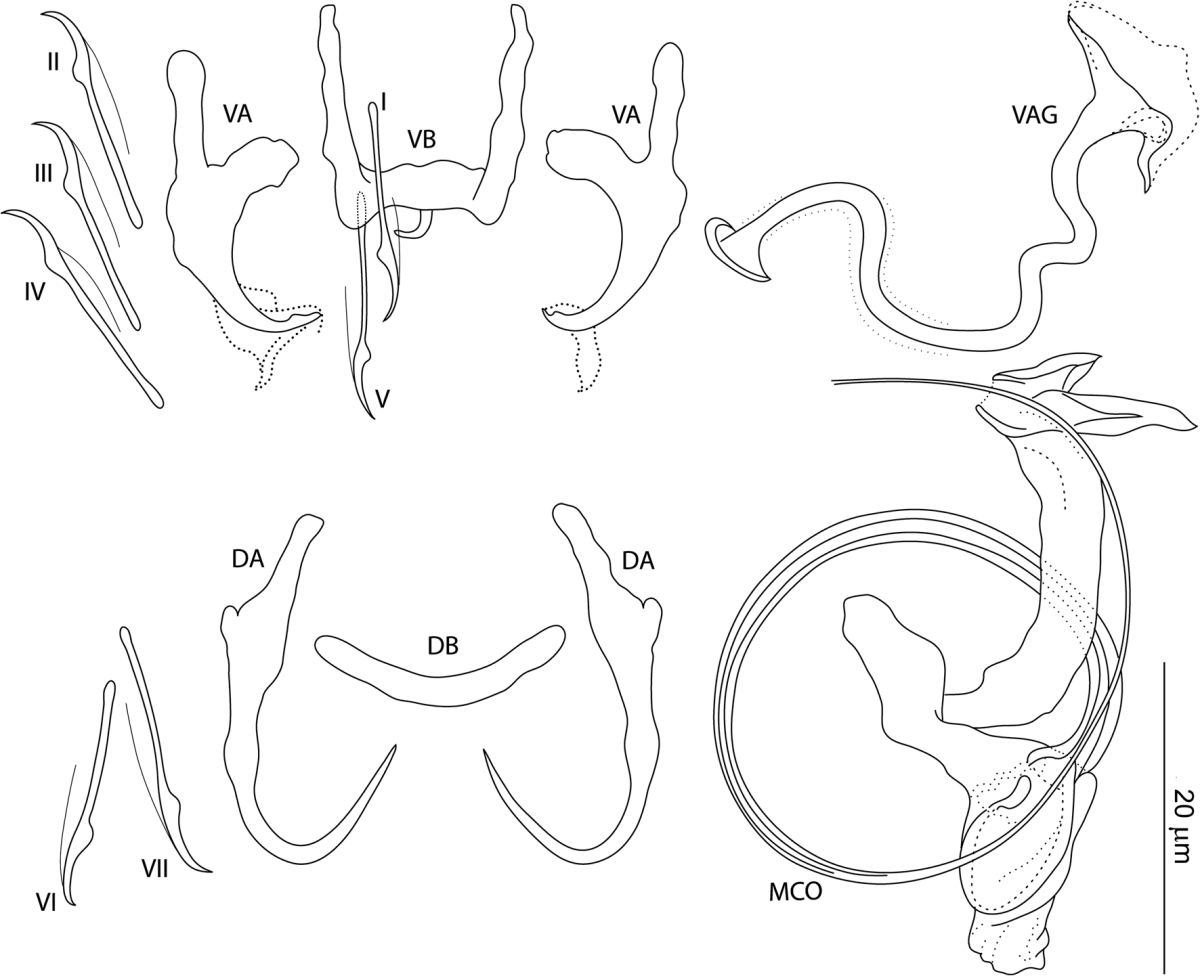
*Characidotrema auritum* Kičinjaová & Řehulková n. sp. Sclerotized structures. *Abbreviations*: VA, ventral anchor; DA, dorsal anchor; VB, ventral bar; DB, dorsal bar; I-VII, hooks; VAG, vagina; MCO, male copulatory organ

[Based on 10 specimens fixed in GAP; Fig. 7]. Ventral anchors with roots nearly perpendicular to each other, evenly arced (recurved) inner root, elongate outer root, poorly differentiated base and shaft, and short scoop-shaped point; anchor filaments poorly differentiated; sheat-like structure usually observed; inner length 10–14 (12; *n* = 10); outer length 17–21 (19; *n* = 10); inner root length 5–6 (5; *n* = 10); outer root length 5–9 (7; *n* = 10); point length 2–4 (3; *n* = 10). Dorsal anchors with elongate inner root, short to reduced outer root, elongate slightly curved shaft with submedial bump-like swelling, and elongate slightly recurved point; anchor filaments often visible; inner length 21–24 (22; *n* = 10); outer length 16–19 (18; *n* = 10); inner root length 7–8 (7; *n* = 10); outer root length 1 (*n* = 10); point length 6–8 (7; *n* = 10). Ventral bar 9–13 (11; *n* = 10) wide; anterior arms 9–14 (11; *n* = 10) long; posteromedial projection 4–5 (4; *n* = 5) long. Dorsal bar broadly V-shaped, 14–18 (16; *n* = 10) wide. Hooks similar; each with undilated shank, poorly developed thumb, 10–15 (13; *n* = 10) long; FH loop 0.5 shank length. Vagina sclerotized, a meandering tube; straight length 10–43 (27; *n* = 10); curved length 29–51 (43; *n* = 10). MCO comprising copulatory tube, accessory piece; total straight length 32–43 (38; *n* = 10). Copulatory tube a coil of about one-and-a-half rings, narrowed distally; base with terminal flange, irregularly shaped robust basal process; tube curved length 109–126 (118; *n* = 10); base length 10–13 (11; *n* = 10); base width 5–7 (6; *n* = 10). Accessory piece articulated to the basal process, massive, C- shaped, with two terminal ear-like branches.

### Molecular characterisation

The combined *18S*-ITS1 sequence of *C. auritum* n. sp. was 973 bp long, of which 483 bp corresponded to the *18S* rDNA and 490 bp corresponded to the ITS1 region. The sequence of partial *28S* rDNA was 772 bp long. Five specimens were sequenced from South Africa and Zimbabwe and no intraspecies variability for the *28S* and *18S*-ITS1 regions was observed.

### Differential diagnosis

*Characidotrema auritum* n. sp. is a sister species to *C. vespertilio* n. sp., according to the similarities of both morphology and DNA sequences. These two new species are probably close to *C. elongata* described from *Brycinus jacksonii* (syn. *Alestes jacksoni*) in Uganda by Paperna & Thurston [5] and later reported on *B. leuciscus* (syn. *Alestes leuciscus*) in Ghana [7] (see also [3]). All three species possess a copulatory tube with a base having a proximal (terminal) flange and a robust basal process to which an accessory piece is attached. However, *C. auritum* n. sp. clearly differs from these two species by possessing a coiled (*vs* sigmoid in *C. vespertilio* and *C. elongata*) and markedly longer copulatory tube (109–126 *vs* 45–51 µm in *C. vespertilio* and 30–40 µm in *C. elongata*). In addition, the vagina of *C. auritum* n. sp. is markedly longer than that of *C. vespertilio* n. sp. (29–51 *vs* 12–19 µm).

***Characidotrema vespertilio*** **Kičinjaová & Řehulková n. sp.**

***Type-host***: *Brycinus imberi* (Peters, 1852).

***Type-locality***: River Boumba **(**03°18′44.28″N, 14°04′40.79″E), Cameroon.

***Other locality***: River Lindi (00°34′38.99″N, 25°07′11.39″E), DR Congo.

***Site on host***: Gill lamellae.

***Type-material***: Holotype, nine paratypes and a hologenophore in IPCAS (M-689).

***Representative DNA sequences***: GenBank: MK012543 (*28S* rDNA) and MK014161 (*18S*-ITS1 rDNA).

***ZooBank registration***: To comply with the regulations set out in article 8.5 of the amended 2012 version of the International Code of Zoological Nomenclature (ICZN) [28], details of the new species have been submitted to ZooBank. The Life Science Identifier (LSID) of the article is urn:lsid:zoobank.org:pub:506A57E6-A1AF-4978-B72D-1C174AAB6F70. The LSID for the new name *Characidotrema vespertilio* Kičinjaová & Řehulková n. sp. is urn:lsid:zoobank.org:act:7993F47D-1CFC-4BB9-B44D-C7C701279ED2.

***Etymology***: The specific epithet (a noun) is from the Latin (*vespertilio* = bat) and refers to the shape of the terminal part of the accessory piece resembling the profile view of a bat’s head.

### Description

**Fig. 8:**
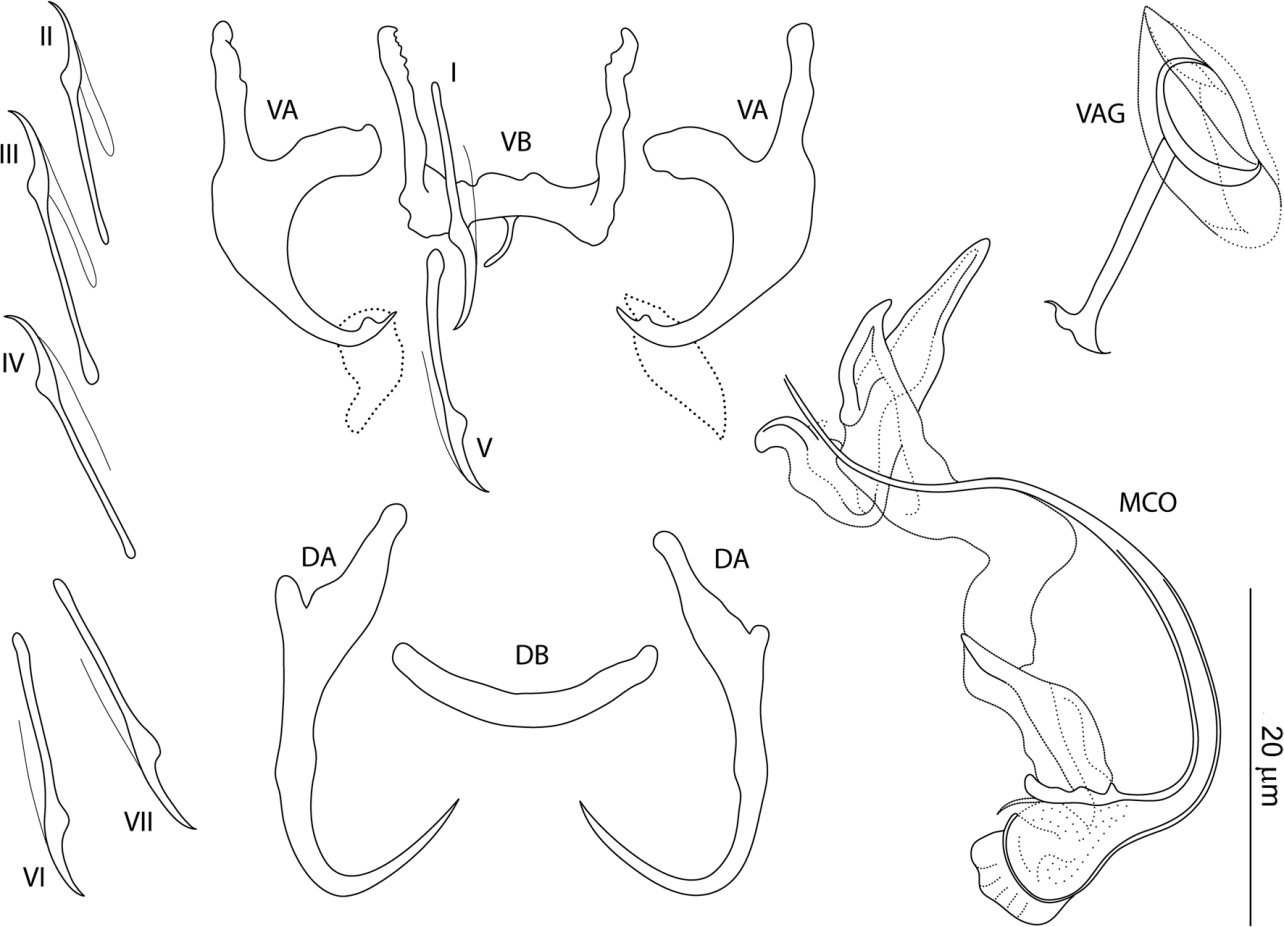
*Characidotrema vespertilio* Kičinjaová & Řehulková n. sp. Sclerotized structures. *Abbreviations*: VA, ventral anchor; DA, dorsal anchor; VB, ventral bar; DB, dorsal bar; I-VII, hooks; VAG, vagina; MCO, male copulatory organ

[Based on 10 specimens fixed in GAP; Fig. 8]. Ventral anchors with roots perpendicular to each other, evenly arced (recurved) inner root, elongate outer root, poorly differentiated base and shaft, and short scoop-shaped point; anchor filaments poorly differentiated; supporting sheath-like structure usually observed; inner length 11–12 (12; *n* = 10); outer length 18–19 (18; *n* = 10); inner root length 5–6 (6; *n* = 10); outer root length 6–8 (7; *n* = 10); point length 2–4 (3; *n* = 10). Dorsal anchors with elongate inner root, short outer root, elongate slightly curved shaft with submedial bump-like swelling, and elongate slightly recurved point; anchor filaments often visible; inner length 22–24 (23; *n* = 10); outer length 17–19 (18; *n* = 10); inner root length 8–9 (8; *n* = 10); outer root length 2 (*n* = 10); point length 8–10 (9; *n* = 10). Ventral bar 10–12 (11; *n* = 10) wide; anterior arms 10–13 (11; *n* = 10) long; posteromedial projection 4–7 (5; *n* = 5) long. Dorsal bar broadly V-shaped, 15–17 (16; *n* = 10) wide. Hooks similar; each with undilated shank, poorly developed thumb, 15–16 (14; *n* = 10) long; FH loop 0.5 shank length. Vagina sclerotized, a straight tube, distal part foliaceous; straight length 16–20 (18; *n* = 10); curved length 12–19 (15; *n* = 10). MCO comprising copulatory tube, accessory piece; total straight length 26–42 (37; *n* = 10). Copulatory tube sigmoid, narrowed distally; base bulbous, with crest shaped basal flange, two-piece basal process; tube curved length 45–51 (48; *n* = 10); base length 8–10 (9; *n* = 10); base width 5–7 (6; *n* = 10). Accessory piece articulated to the basal process, massive, L-shaped, with three terminal branches (two ear-like and one claw-like).

### Molecular characterization

The combined *18S*-ITS1 sequence of *C. vespertilio* n. sp. was 972 bp long, of which 483 bp corresponded to the *18S* rDNA and 489 bp corresponded to the ITS1 region. The sequence of partial *28S* rDNA was 761 bp long. A total of 10 specimens were sequenced from D.R Congo and Cameroon and no intraspecies genetic variability was observed in any marker.

### Differential diagnosis

This new species is similar to *C. auritum* n. sp. and *C. elongata* Paperna & Thurston, 1968 in having an accessory piece attached to a robust basal process of the copulatory tube. Features distinguishing *C. vespertilio* n. sp. from *C. auritum* n. sp. are given in the differential diagnosis for the latter species. *Characidotrema vespertilio* n. sp. differs from *C. elongata* by possessing a more robust accessory piece with the distal end formed as three prominent branches (*vs* distal end cheliform, less prominent in *C. elongata*).

### Genetic relationships among *Characidotrema* spp.

No intraspecific sequence variation was observed between specimens of the same species of *Characidotrema*. Values of pairwise genetic comparisons are shown in Table 5. The uncorrected p-distances among six *Characidotrema* spp. ranged between 0–0.019 for *18S*, 0.024–0.218 for ITS1 and 0.004–0.059 for *28S*, while the average interspecific p-distances were 0.011, 0.174 and 0.046 for *18S*, ITS1 and *28S*, respectively. The smallest interspecific distances were observed between *C. auritum* n. sp. and *C. vespertilio* n. sp. for each marker, both infecting *B. imberi*. *Characidotrema spinivaginus* was revealed as the most genetically distant species to *C. auritum* n. sp. or *C. vespertilio* n. sp. (0.019) for *18S*, to *C. brevipenis* (0.218) for ITS1, and to *C. pollex* n. sp. (0.059) for *28S*.

**Table 5:** The uncorrected p-distances (below the diagonal) and nucleotide differences (above the diagonal) among *18S*, ITS1 and *28S* rDNA sequences of *Characidotrema* spp. studied

		1	2	3	4	5	6
*18S*							
1	*C. auritum* n. sp	–	6	6	5	9	0
2	*C. brevipenis*	0.012	–	2	5	7	6
3	*C. nursei*	0.012	0.004	–	5	7	6
4	*C. pollex* n. sp.	0.010	0.010	0.010	–	4	5
5	*C. spinivaginus*	0.019	0.014	0.014	0.008	–	9
6	*C. vespertilio* n. sp.	0.000	0.012	0.012	0.010	0.019	–
ITS1							
1	*C. auritum* n. sp.	–	71	57	77	70	9
2	*C. brevipenis*	0.191	–	35	77	81	73
3	*C. nursei*	0.153	0.094	–	69	64	62
4	*C. pollex* n. sp.	0.207	0.207	0.185	–	80	77
5	*C. spinivaginus*	0.188	0.218	0.172	0.215	–	70
6	*C. vespertilio* n. sp.	0.024	0.196	0.167	0.207	0.188	–
*28S*							
1	*C. auritum* n. sp.	–	36	31	37	36	3
2	*C. brevipenis*	0.049	–	18	36	38	37
3	*C. nursei*	0.042	0.025	–	38	41	34
4	*C. pollex* n. sp.	0.051	0.049	0.052	–	43	40
5	*C. spinivaginus*	0.049	0.052	0.056	0.059	–	39
6	*C. vespertilio* n. sp.	0.004	0.051	0.046	0.055	0.053	–

### Phylogenetic placement of *Characidotrema* spp. within African dactylogyrids based on *28S* rDNA sequences

**Fig. 9:**
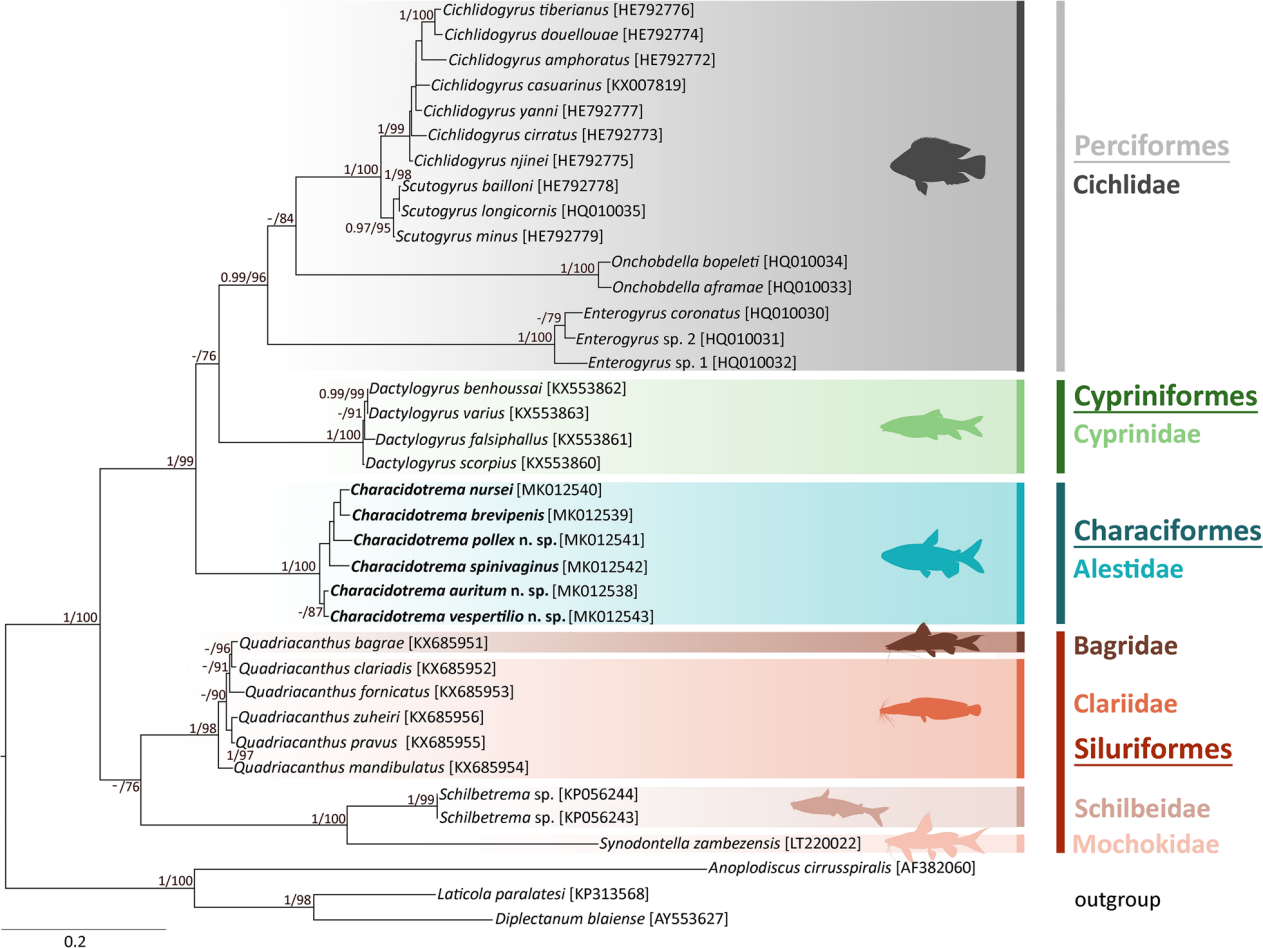
Maximum Likelihood tree inferred from the analysis of *28S* rDNA sequences including 34 selected species of African Dactylogyridae. Values along branches indicate posterior probabilities and boostrap values resulting from Bayesian Inference (BI) and Maximum Likelihood (ML) analyses. Only values > 0.95 for BI and > 70% for ML are shown. The phylogenetic tree was rooted using three species belonging to the Anoplodiscidae (*Anoplodiscus cirrusspiralis* Roubal, Armitage & Rohde, 1983) and the Diplectanidae (*Diplectanum blaiense* Gupta & Khanna, 1974 and *Laticola paralatesi* (Nagibina, 1976) Yang, Kritsky, Sun, Zhang, Shi & Agrawal, 2006)

BI and ML analyses produced trees with similar topologies for the African Dactylogyridae included in the analyses. The final tree based on ML phylogenetic analysis is given in Fig. 9. African dactylogyrids formed two strongly supported clades: (i) dactylogyrids of catfishes (Siluriformes) consisting of lineages infecting bagrids (*Q. bagrae* Paperna, 1979), clariids (*Quadriacanhus* Paperna, 1961), schilbeids (*Schilbetrema* Paperna & Thurston, 1968) and mochokids (*Synodontella* Dossou & Euzet, 1993); and (ii) parasites of cichlids (*Cichlidogyrus* Paperna, 1960, *Scutogyrus* Pariselle & Euzet, 1995, *Onchobdella* Paperna, 1968 and *Enterogyrus* Paperna, 1963), cyprinids (*Dactylogyrus* Diesing, 1850), and alestids (*Characidotrema*). Species of *Characidotrema* formed a highly supported group by both BI and ML analyses (Fig. 9). Likewise, the remaining dactylogyrid genera were revealed as monophyletic groups well supported by both analyses. Relationships between species parasitizing Cypriniformes and Perciformes were only supported by ML analysis.

## Discussion

Species of *Characidotrema* are known only from the gills of African tetras (Alestidae), although the majority of them occur on species of *Brycinus*, a speciose Pan-African genus including commercially important species. Of the 13 valid species in *Characidotrema* (including the new species described herein), ten have been recorded only from *Brycinus* spp., two species from hosts representing both *Brycinus* and *Alestes*, and only one species from hosts belonging to *Hemigrammopetersius* Pellegrin and *Phenacogrammus* Eigenmann (see Table 1).

**Fig. 10:**
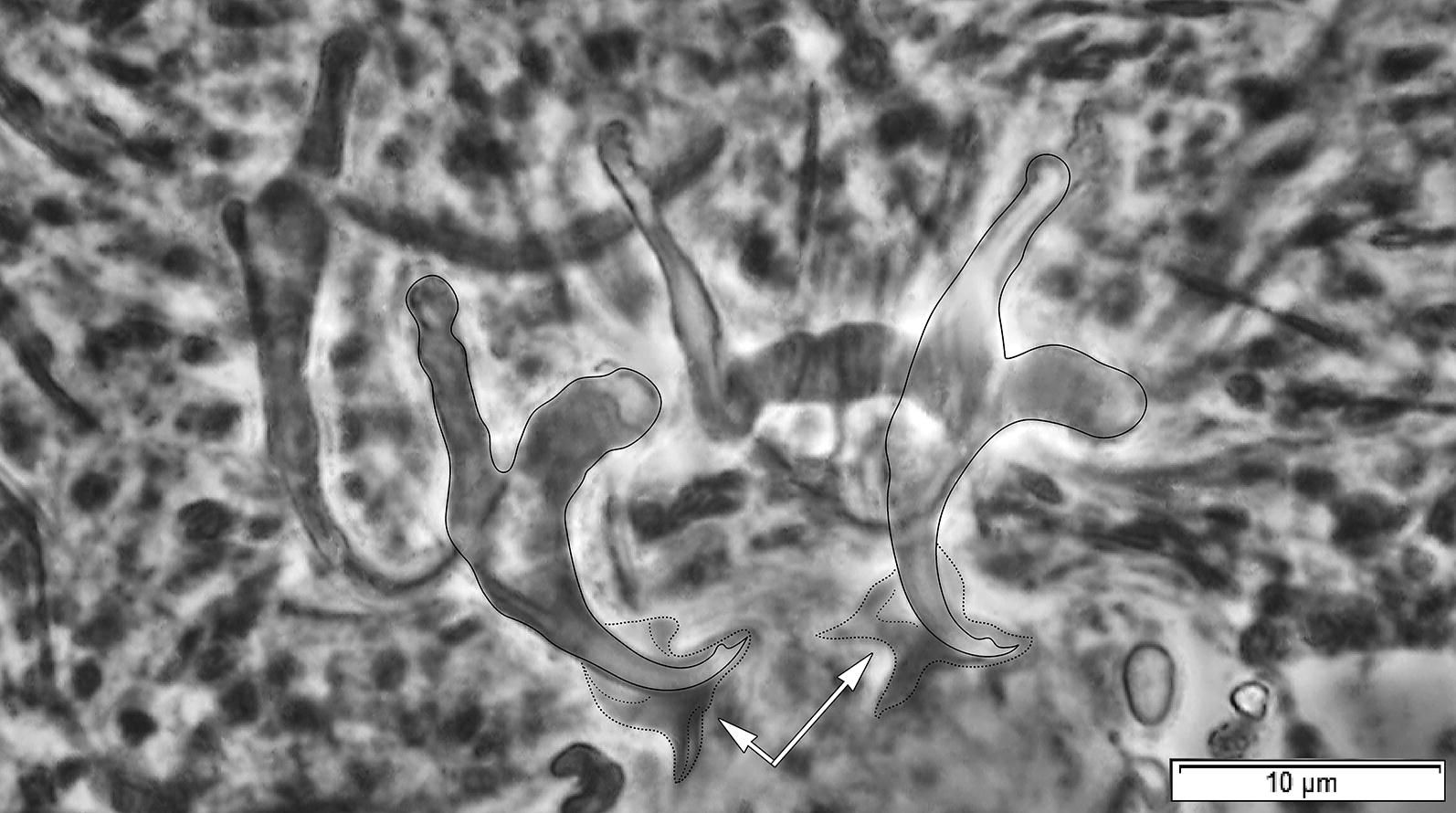
Haptoral sclerotized elements of *Characidotrema brevipenis* Paperna, 1969 showing the sheath-like structure associated with the shaft and point of the ventral anchors (Phase-contrast micrograph combined with line drawings)

All members of *Characidotrema* including the new species described herein possess a relatively uniform configuration and morphology of the haptoral sclerites [3]. The dorsal anchors, characterized by a submedially swollen shaft, are morphologically (in shape) indistinguishable among species of *Characidotrema*; therefore, they are not suitable for species determination. Additionally, the shape of the highly modified ventral anchors is also very similar in all species of the genus. However, the shape of their roots slightly varies among specimens of individual species of *Characidotrema* and even between the ventral anchors of one and the same specimen (see Figs. 2, 3, 4, 5, 6, 7, 8), although the length of the roots is relatively constant (i.e. species-specific). The diagonally truncate or scoop-shaped point of the ventral anchor represents an unusual feature that is unique among African dactylogyrids [3]. Our observations on the ventral anchors of *Characidotrema* spp. by means of phase contrast microscopy revealed the presence of a sheath-like structure associated with the shaft and point of the ventral anchors (Fig. 10). Molnár & Mossalam [10] were the only authors who described and illustrated such a structure before the present study. However, it should be mentioned that, since these structures are not (or only weakly) sclerotized, they were not always visible in all specimens of the species of *Characidotrema* in the present collection. A similar feature has previously been reported for species of *Triacanthinella* Bychowsky & Nagibina, 1968 (Dactylogyridae) from triacanthid teleosts of Peninsular Malaysia [29]. However, in *Triacanthinella* spp., a structure associated with the lower portion of the anchors is formed as a sheath-like sclerite closely enveloping the shaft and point of the anchors, whereas in the present species this structure appears to be more independent, with a distal part resembling a caudal fin. Unfortunately, it is impossible, without studying the haptoral armament using more sophisticated techniques (e.g. laser scanning confocal fluorescence microscopy), to suggest the function of these structures. The ventral bar in *Characidotrema* spp. appears to be flexible, resulting in variability in its shape in fixed specimens. The hooks basically exhibit the typical “ancyrocephaline” distribution described by Mizelle [14] for dactylogyrids. However, in all specimens studied here, pairs I and V are placed in close proximity (at the level) of the ventral bar (pair I lies more anteriorly, i.e. between the arms of the bar), whereas in other dactylogyrids these pairs are usually situated further anteriorly (pair I) and posteriorly (pair V) from the ventral bar, i.e. further apart from each other. The same arrangement of hooks is also observed in the original drawings of the haptoral configuration given for the following previously described species of *Characidotrema*: *C. brevipenis* [3], *C. regia* [11], *C. spiropenis* Birgi, 1988 [11] and *C. undifera* [3]. It seems highly probable, although not mentioned or figured in their original descriptions, that this hook distribution occurs in all previously described species of *Characidotrema*.

**Fig. 11:**
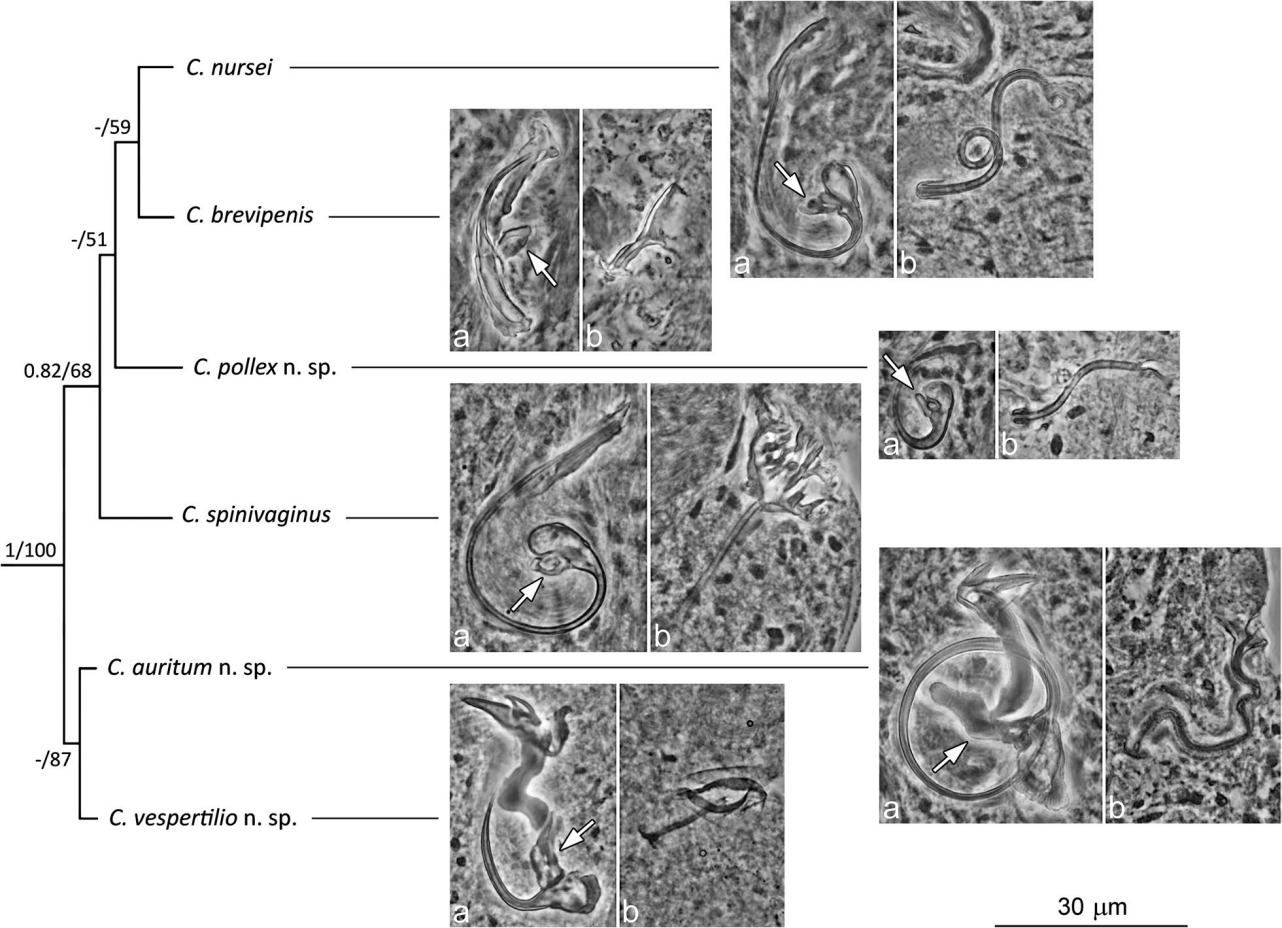
Maximum Likelihood tree showing the division of *Characidotrem*a spp. in accordance with the morphology of the MCO (**a**) and vagina (**b**). Arrows indicate one of the principal characters dividing *Characidotrema* spp. into two morphological groups

As indicated above, the most apparent character distinguishing species in the genus is the morphology of the MCO and vagina. In the case of the MCO, two morphological types dividing *Characidotrema* spp. in the present collection into morphological groups may be defined. The first group includes five species (*C. brevipenis*, *C. nursei*, *C. pollex* n. sp., *C. spinivaginus* and *C. zelotes*) with an MCO composed of a copulatory tube with a finger-like basal process rising (at different angles) from the distal part of the base, and an accessory piece mostly not articulated to the base (if articulated, then by a poorly sclerotized part as in *C. brevipenis*; Figs. 2, 11). The second group, including two new species (*C. auritum* n. sp. and *C. vespertilio* n. sp.), is characterized by a copulatory tube with a crest-shaped terminal flange and a robust basal process to which an accessory piece is articulated.

The division into two morphological groups was also supported by the results of phylogenetic analyses based on *28S* rDNA sequences (Fig. 11). The ML phylogenetic reconstruction showed that *Characidotrema auritum* n. sp. from *B. imberi* clusters together with *C. vespertilio* n. sp. from the same host. This clade was well separated from the clade of *Characidotrema* spp. found on *Brycinus nurse*. Thus, the basic structure of the MCO (along with host preferences) appears to be the best morphological tool for resolving relationships among species of *Characidotrema*. This result supports the MCO type concept [30] suggesting that the morphology of the MCO carries much information of systematic/phylogenetic importance and that species with a particular MCO type often parasitize a particular major host taxon [13, 31, 32]. However, *C. ruahae* (Paperna, 1979) from *B. imberi* is morphologically more similar to *C. brevipenis* and *C. nursei* from *B. nurse* [3] than to the two new species from *B. imberi*. Although we cannot verify the accuracy of the host identification, it could be interesting to resolve the phylogenetic relationships between these *Characidotrema* spp. possessing similar morphological characters of the MCO but parasitizing different host species. Conversely, in terms of the haptor, all thirteen species of *Characidotrema* considered valid exhibit a relatively uniform morphology of the haptoral sclerites. Considering that similar attachment organs will lead to the choice of the same microhabitats on the host, as postulated by Rohde [33], then it can be assumed that all currently known species of *Characidotrema* would occupy the same niche on the gills of their hosts. In addition, as indicated in Table 1, communities of *Characidotrema* spp. on respective hosts can display a relatively high species richness, which ranges from one (e.g. on *B. kingsleyae*) to six (reported on *B. nurse*) species per host species. Our study revealed that up to three species, *C. nursei*, *C. pollex* n. sp. and *C. spinivaginus* or *C. brevipenis*, *C. spinivaginus* and *C. zelotes*, may simultaneously parasitize *B. nurse*. The coexistence of several species of *Characidotrema* on the same host, together with the narrow interspecific variability in the haptoral sclerites and the comparatively wider interspecific variability in the MCOs, supports the idea that parasite species occupying the same microhabitats are reproductively isolated by morphological differentiation of their copulatory organs that probably avoids the hybridization [34–36]. Our results also suggest that the morphology of the MCO is not the only factor affecting reproductive isolation among *Characidotrema* spp. The morphological differences of the vagina, associated with the degree of sclerotization, probably also play an important role in the reproductive isolation of these species. On the other hand, due to the high variability of this organ even among closely related species, it appears that the morphology of the vagina can make only a minimal contribution with respect to clarifying relationships among species of this genus.

Phylogenetic analyses based on *28S* rDNA sequences supported the monophyly of *Characidotrema* spp., and indicated the closer relationship of this genus to monogeneans parasitizing African cyprinids (*Dactylogyrus* spp.) and cichlids (species of *Cichlidogyrus*, *Scutogyrus* and *Onchobdella*) than to those parasitizing catfishes (species of *Quadriacanthus*, *Schilbetrema* and *Synodontella*). Our results indicate that *Characidotrema* spp. from the same host are more closely related and suggest possible intra-host speciation.

The majority of *Characidotrema* spp. are restricted to species of *Brycinus*, which suggests a long association between these parasites and the host genus. Recent molecular studies on *Brycinus* species have repeatedly indicated that this taxon is polyphyletic [37–40] and support the monophyly of the “macrolepidotus” species group of Paugy [41] comprising eight large-bodied species (including *B. macrolepidotus* Valenciennes, the type-species of the genus) mainly distributed in Central Africa. Phylogenetic analyses presented by Arroyave & Stiassny [40] placed *B. imberi* and *B. nurse* together in a clade of *Brycinus* (*sensu lato*) recovered as sister to a group of species belonging to *Bryconaethiops* Günther. Parasites, especially highly host-specific monogeneans, are usually considered to be good biological markers of their host diversity and evolution [42, 43]. However, current knowledge on the diversity and distribution of *Characidotrema* spp. is insufficient for either supporting or rejecting the above hypotheses on *Brycinus* phylogeny. A total of 13 species of *Characidotrema* and 19 species of *Annulotrema* are known from five and seven species of *Brycinus*, respectively. Considering that 36 species of *Brycinus* are currently recognized as valid (FishBase [12]), the diversity of monogeneans parasitizing these fishes appears to be poorly understood.

## Conclusions

The present study provides first insights into the molecular phylogeny of monogeneans parasitizing African tetras. Morphological and molecular data suggest *Characidotrema* to have a monophyletic nature and indicate the closer relationship of this genus to monogeneans parasitizing African cyprinids and cichlids than to those parasitizing catfishes. The overall agreement between the morphological diversification of the MCOs and the molecular tree generated in this study indicates that significant phylogenetic signal for clarifying relationships among species of *Characidotrema* is associated with certain characteristics of the MCO. The present study suggests that intra-host speciation is probably one of the important ecological processes involved in species diversification in *Characidotrema*. Nevertheless, further morphological and molecular samplings of monogeneans of both genera, *Characidotrema* and *Annulotrema*, are needed to elucidate questions related to the ecological similarity, coexistence, and phylogeny of these parasites. To identify potential co-speciation events, co-phylogenetic analyses of these monogeneans and their alestid hosts are required.

## Data Availability

The data supporting the results of this study are included within the article. The type- and voucher material is deposited in the Institute of Parasitology, Czech Academy of Science, České Budějovice, Czech Republic under the accession numbers IPCAS (M-282, M-686–M-691). The newly generated sequences were submitted to the GenBank database under the accession numbers MK014156–MK014161 (*18S*-ITS1) and MK012538–MK012543 (*28S*).

## Abbreviations

BI: Bayesian inference
BPP: Bayesian posterior probability
GAP: glycerine and ammonium picrate medium
IPCAS: Institute of Parasitology, Biology Centre of the Czech Academy of Sciences
ITS1: internal transcribed spacer 1
MCO: male copulatory organ
MCMC: Markov Chain Monte Carlo
ML: maximum likelihood
MRAC: Musée Royal de lʼAfrique Centrale
PCR: Polymerase chain reaction
